# Deep learning for cardiovascular management: optimizing pathways and cost control under diagnosis-related group models

**DOI:** 10.3389/frai.2025.1580445

**Published:** 2025-09-01

**Authors:** Haohao Chen, Ying Zeng, De Cai

**Affiliations:** ^1^Department of Pharmacy, The First Affiliated Hospital of Shantou University Medical College, Shantou, China; ^2^Department of Pharmacology, Shantou University Medical College, Shantou, China

**Keywords:** diagnosis-related group, cardiovascular disease management, deep learning, clinical pathway, predictive analytics in healthcare, AI-driven healthcare solutions

## Abstract

Cardiovascular diseases (CVDs) remain the leading causes of morbidity, mortality, and healthcare expenditures, presenting substantial challenges for hospitals operating under Diagnosis-Related Group (DRG) payment models. Recent advances in deep learning offer new strategies for optimizing CVD management to meet cost control objectives. This review synthesizes the roles of deep learning in CVD diagnosis, treatment planning, and prognostic modeling, emphasizing applications that reduce unnecessary diagnostic imaging, predict high-cost complications, and optimize the utilization of critical resources like ICU beds. By analyzing medical images, forecasting adverse events from patient data, and dynamically optimizing treatment plans, deep learning offers a data-driven strategy to manage high-cost procedures and prolonged hospital stays within DRG budgets. Deep learning offers the potential for earlier risk stratification and tailored interventions, helping mitigate the financial pressures associated with DRG reimbursements. Effective integration requires multidisciplinary collaboration, robust data governance, and transparent model design. Real-world evidence, drawn from retrospective studies and large clinical registries, highlights measurable improvements in cost control and patient outcomes; for instance, AI-optimized treatment strategies have been shown to reduce estimated mortality by 3.13%. However, challenges—such as data quality, regulatory compliance, ethical issues, and limited scalability—must be addressed to fully realize these benefits. Future research should focus on continuous model adaptation, multimodal data integration, equitable deployment, and standardized outcome monitoring to validate both clinical quality and financial return on investment under DRG metrics. By leveraging deep learning’s predictive power within DRG frameworks, healthcare systems can advance toward a more sustainable model of high-quality, cost-effective CVD care.

## 1 Introduction

Cardiovascular diseases (CVDs) remain a leading cause of morbidity and mortality globally, accounting for significant healthcare expenditures. The management of CVD often involves complex diagnostic and clinical pathways—structured, evidence-based, multidisciplinary care plans designed to manage specific clinical conditions, including advanced imaging, invasive procedures, and prolonged hospital stays (Roth et al., 2020; Benjamin et al., 2019; Figtree et al., 2023). These resource-intensive interventions pose substantial challenges for cost control, especially in healthcare systems adopting Diagnosis-Related Group (DRG) payment models. DRG systems aim to standardize payments by classifying hospital cases with similar clinical characteristics and resource consumption (Busse et al., 2013). This standardization motivates healthcare providers to deliver care within specified cost parameters while ensuring acceptable patient outcomes, a balance that requires aligning clinical practices with DRG benchmarks without compromising care quality (Milstein and Schreyogg, 2024; Kutz et al., 2019). It is important to note, however, that DRG systems are not monolithic; their structure, payment formulas, and clinical impact vary significantly across countries. This review primarily focuses on the challenges and optimization opportunities within the established DRG frameworks typical of high-income countries, such as those in the United States, Europe, and Australia (Milstein and Schreyogg, 2024).

The complexity of CVD management arises not only from the diversity of clinical presentations but also from the variability in resource utilization across healthcare providers. For example, differences in diagnostic preferences, treatment modalities, and postoperative care protocols contribute to significant cost variations. This inconsistency often leads to challenges in meeting DRG payment standards, as hospitals may struggle to maintain financial sustainability while delivering high-quality care. Additionally, the increasing prevalence of CVDs due to aging populations and lifestyle-related risk factors exacerbates the economic burden on healthcare systems (Virani et al., 2021).

Recent advancements in deep learning have opened new avenues for optimizing clinical pathways in CVD management (Attia et al., 2019; Esteva et al., 2019). By leveraging large-scale patient data (such as from electronic health records, clinical registries, and imaging databases), deep learning algorithms can identify inefficiencies, predict patient outcomes, and recommend cost-effective interventions tailored to individual cases. These capabilities are particularly valuable for addressing the disparities between actual treatment costs and DRG payment standards. For instance, predictive models can flag patients at risk of prolonged hospital stays or costly complications, enabling proactive adjustments to their care plans. Moreover, deep learning tools can facilitate personalized medicine by integrating multi-modal data, such as clinical records, imaging results, and genetic information, to develop precise and efficient treatment strategies (Esteva et al., 2019).

The integration of deep learning into CVD management also supports decision-making for clinicians by providing real-time insights and evidence-based recommendations. These tools can enhance diagnostic accuracy, streamline workflows, and improve resource allocation. For example, convolutional neural networks (CNNs) have been used to analyze cardiac imaging, while recurrent neural networks (RNNs) excel in modeling sequential data, such as patient histories. Reinforcement learning algorithms further enable dynamic adjustments to clinical pathways, optimizing treatment sequences to balance costs and outcomes (Lundervold and Lundervold, 2019; Chen et al., 2022).

In this narrative review, we explore the intersection of deep learning and DRG-aligned clinical pathway optimization for cardiovascular diseases. It synthesizes recent applications of deep learning in CVD diagnosis, treatment planning, and cost management, highlighting their potential to transform healthcare delivery. Furthermore, it identifies current challenges, such as data quality issues, model interpretability, and integration into clinical workflows, and discusses future research directions. To synthesize the current evidence, this review draws upon literature from major scientific databases, including PubMed and Google Scholar, focusing on key publications and developments in the field. By addressing these gaps, deep learning-driven approaches can contribute to sustainable and equitable healthcare systems that balance financial constraints with clinical excellence.

## 2 Applications of deep learning in cardiovascular disease management

Deep learning has significantly transformed CVD management by improving diagnostic accuracy, optimizing treatment planning, and enabling effective prognostic modeling. These applications harness the power of artificial intelligence to address key challenges in clinical practice, such as the complexity of CVD presentations and the need for cost-effective care.

### 2.1 Diagnostic support

Deep learning has significantly enhanced diagnostic precision in CVD management. CNNs, known for their exceptional performance in image analysis, function much like a digital microscope trained to see specific patterns. They automatically learn to identify features ranging from simple lines and textures to complex anatomical structures like cardiac chambers, making them ideally suited for analyzing cardiac imaging modalities, including echocardiograms, cardiac MRI, and CT scans (Lundervold and Lundervold, 2019; Rajpurkar et al., 2017). These models can segment cardiac structures with precision, enabling the detection of structural abnormalities and early signs of diseases like cardiomyopathy. This segmentation facilitates accurate assessment of heart function and supports diagnostic outcomes comparable to those of expert cardiologists. For example, CNN-based segmentation approaches have demonstrated high accuracy in distinguishing healthy from pathological cardiac structures, aiding in early diagnosis and treatment planning (Song et al., 2022).

RNNs, particularly architectures like long short-term memory (LSTM) networks, are designed with a form of memory. This allows them to process sequential data, such as an ECG signal over time, by considering previous data points when interpreting a new one. This “memory” makes them highly effective in detecting arrhythmias from ECG data, as they can learn the time-dependent patterns of a heartbeat (Bjerken et al., 2023; Adedinsewo et al., 2020; Pokaprakarn et al., 2022; Laghari et al., 2023). By analyzing time-series patterns, these models can identify irregular heart rhythms, such as atrial fibrillation and ventricular tachycardia, withhigh sensitivity and specificity, achieving performance comparable to that of expert cardiologists and, in some cases, classification accuracies exceeding 97% (Nagarajan et al., 2021; Cinar and Tuncer, 2021).

### 2.2 Treatment planning

Treatment planning for CVDs often requires tailoring interventions to individual patient needs. Deep learning models, particularly reinforcement learning algorithms, optimize treatment regimens by balancing efficacy and cost-effectiveness (Kadem et al., 2023). These algorithms utilize real-time patient data to suggest precise medication adjustments, ensuring therapeutic goals are achieved while minimizing potential side effects and reducing healthcare costs. Examples include optimizing drug dosing in critical care and managing chronic conditions with dynamic, personalized interventions (Liu et al., 2020).

Predictive models also play a pivotal role in planning invasive procedures. Machine learning algorithms leverage clinical and imaging data to estimate the likelihood of success for interventions like stent placement or coronary artery bypass grafting (CABG) (Bertsimas et al., 2020; Ninomiya et al., 2023). By integrating patient demographics, comorbidities, and procedural factors, these models enable clinicians to make informed decisions about the most appropriate treatment approach. Such precision enhances patient outcomes and supports tailored clinical strategies (Li et al., 2024).

### 2.3 Prognostic modeling

Prognostic modeling is essential for stratifying patients based on their risk profiles and planning long-term care strategies. LSTM networks, a type of RNN, have proven highly effective in analyzing time-series data to predict outcomes such as heart failure, myocardial infarction, and rehospitalization risks (Lu et al., 2021; Chu et al., 2020; Baral et al., 2021; Rai and Chatterjee, 2021). By incorporating dynamic patient variables, including lab results, vital signs, and medication history, these models deliver precise and individualized risk assessments (Chi et al., 2021; Yu and Son, 2024).

In addition to enabling granular risk stratification, deep learning algorithms facilitate population-level analyses, revealing trends and patterns within extensive datasets. These insights are instrumental in optimizing resource allocation, ensuring that high-risk patients receive timely and intensive care while minimizing unnecessary interventions for low-risk individuals (Bates et al., 2014).

**Figure 1:**
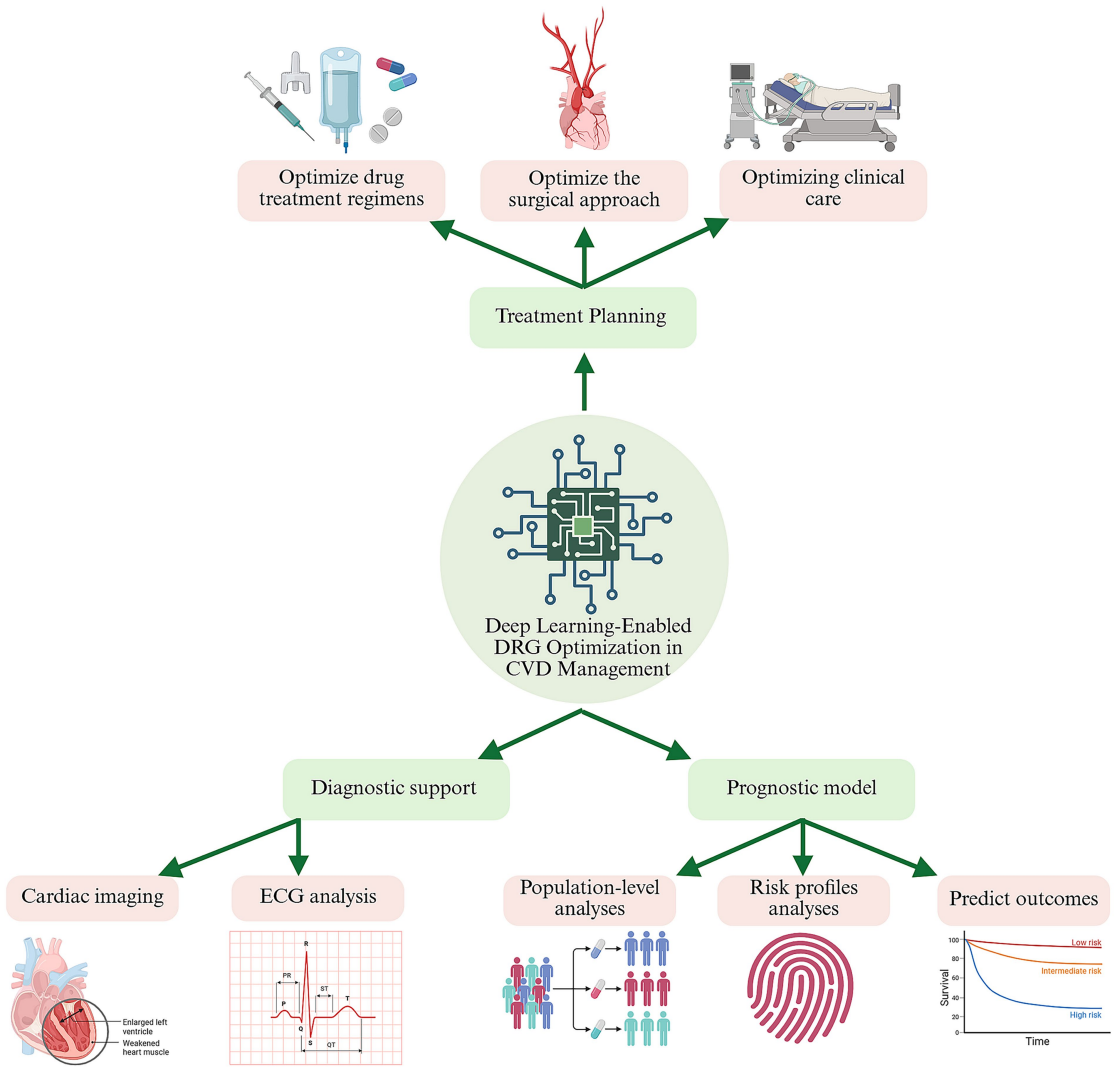
Deep-learning decision support across the cardiovascular-care continuum under diagnosis-related-group (DRG) payment constraints. A central deep-learning engine ingests multimodal data—cardiac images, ECGs, electronic health records, and population registries—to generate three tiers of intelligence: Treatment planning (top branch). Data-driven optimisation of drug regimens, procedural strategy, and peri-operative workflows, maximising clinical benefit while staying within DRG episode budgets. Diagnostic support (lower left). CNN-and LSTM-based tools enhance image and rhythm interpretation, enabling earlier, more accurate diagnoses and improving DRG coding fidelity. Prognostic modelling (lower right). Recurrent, graph, or hybrid networks stratify risk and predict outcomes (e.g., 30-day readmission), guiding targeted follow-up and resource allocation. Green arrows indicate the continuous feedback loop whereby real-world outcomes update model parameters, progressively aligning quality of care with DRG cost goals. CVD, cardiovascular disease; ECG, electrocardiogram BioRender.com.

The integration of deep learning into CVD management holds immense potential for improving clinical outcomes while addressing cost constraints imposed by DRG systems. By enhancing diagnostic precision, tailoring treatments, and enabling proactive care, these technologies contribute to a more efficient and patient-centered healthcare system. Figure 1 provides a schematic representation of the application of deep learning in CVD management.

## 3 DRG requirements and cost challenges in CVD management

### 3.1 Key features of DRG systems

The DRG system is a prevalent hospital payment model designed to enhance both cost efficiency and quality of care (Goldfield, 2010; Mihailovic et al., 2016; Yuan et al., 2019; Zou et al., 2020). Under this system, hospital cases are categorized based on clinical similarities and resource utilization, with each category assigned a fixed payment rate. This standardization motivates healthcare providers to deliver care within specified cost parameters while ensuring acceptable patient outcomes (Goldfield, 2010; Mihailovic et al., 2016).

The DRG systems promote cost-effective care by correlating payments with clinical complexity. For instance, more intricate procedures, such as multi-vessel CABG, receive higher reimbursement rates compared to simpler interventions like single-vessel angioplasty (Varani et al., 2005; Spadaccio and Benedetto, 2018). An Italian study revealed that while the costs for single-vessel percutaneous coronary intervention (PCI) generally match DRG reimbursement levels, multi-vessel PCI costs surpass the DRG rate by approximately 40%. This differentiation ensures that hospitals are adequately compensated for more resource-intensive procedures while discouraging unnecessary interventions (Varani et al., 2005; Spadaccio and Benedetto, 2018).

Furthermore, the DRG framework emphasizes quality outcomes by limiting the overuse of services, thereby reducing wasteful spending and enabling hospitals to reallocate resources toward high-value care (Zou et al., 2020; Spadaccio and Benedetto, 2018). The DRG systems encourage hospitals to minimize the length of stay and the use of ancillary services without compromising patient outcomes (Zou et al., 2020). Achieving this balance, however, requires precise alignment of clinical practices with DRG benchmarks, a challenge given the variability in patient presentations and treatment requirements (Goldfield, 2010; Yuan et al., 2019).

Beyond cost efficiency, The DRG systems enhance resource management within hospitals by integrating clinical and financial aspects of care through a unified framework. This integration improves transparency and provides a solid foundation for assessing both care processes and their financial implications (Zou et al., 2020). The structured nature of DRGs has also spurred innovations such as clinical pathways and DRG-specific management tools, further optimizing operational efficiency and care quality (Mihailovic et al., 2016).

### 3.2 CVD-specific cost drivers

Managing CVD within the DRG framework presents distinct challenges due to the high costs of interventions and the variability in patient outcomes. The primary cost drivers in CVD management include:

High-Cost Procedures: Invasive interventions such as PCI and CABG represent a substantial portion of CVD-related expenditures. These procedures necessitate advanced technologies, specialized healthcare professionals, and extended operating times, all of which contribute to their elevated costs. For example, while CABG offers superior long-term outcomes in complex cases, it incurs significantly higher initial costs due to the need for surgical collateralization and sophisticated equipment (Doenst et al., 2019; Gholami et al., 2019). A systematic review highlighted that the incremental cost-effectiveness ratios (ICERs) for CABG compared to medical therapy range from $4,403 in Brazil to $212,800 in the USA, emphasizing its economic impact (Gholami et al., 2019).

Prolonged Hospital Stays: Complications such as postoperative infections, arrhythmias, and exacerbations of heart failure frequently extend hospital stays. Postoperative atrial fibrillation, occurring in approximately 20–40% of CABG patients, can add an average of $10,000–$20,000 to hospital costs and extend the length of stay by 2–5 days (Greenberg et al., 2017; Gudbjartsson et al., 2020). Additionally, surgical site infections, which affect up to 19% of cardiac surgery patients, significantly escalate both treatment costs and hospitalization duration (Schweizer et al., 2015; Miranda et al., 2021). Readmissions due to heart failure within 30 days also contribute considerably to overall costs, with risk factors including extended hospital stays, chronic kidney disease, and arrhythmias (Sabe et al., 2023; El-Chami et al., 2012). These extended admissions often result in costs that surpass standard DRG reimbursements, presenting financial challenges for hospitals.

Frequent Utilization of Advanced Diagnostics and Therapies: The dependence on sophisticated imaging techniques, such as cardiac MRI and CT angiography, alongside the adoption of novel pharmacological therapies, drives substantial costs in CVD management. Advanced imaging modalities are essential for precise diagnostics and enhanced patient outcomes; however, their high costs pose significant financial hurdles. For instance, the increasing use of coronary CT angiography has led to notable rises in healthcare spending, despite improvements in cardiovascular outcomes and reductions in mortality rates (Centonze et al., 2020; Weir-McCall et al., 2023). Similarly, innovative therapies, including those informed by pharmacogenomics, improve treatment accuracy but introduce financial variability. A systematic review of pharmacogenomics in cardiovascular treatments revealed mixed results regarding cost-effectiveness, depending on specific drug-gene interactions and the context of different health systems (Zhu et al., 2020). These advancements, while beneficial for patient care, challenge hospitals to operate within the financial constraints imposed by DRG payment structures.

### 3.3 Challenges in compliance

Despite the DRG system’s emphasis on efficiency, implementing CVD management strategies that align with DRG standards can be challenging. Hospitals frequently encounter the following barriers:

Variability in Patient Responses: Patient outcomes are heavily influenced by comorbidities, genetic factors, and adherence to treatment plans, making standardized care pathways difficult to implement. For instance, epigenetic factors and conditions such as diabetes or hypertension can significantly affect the recovery process following myocardial infarction. Such diversity in patient presentations complicates cost forecasting and hampers the consistent delivery of care (Damluji et al., 2021; Lee et al., 2020; Schiano et al., 2020).

Resource Allocation: Balancing limited healthcare resources between high-cost and lower-cost cases is an ongoing concern. Overallocating resources to specific cases can strain budgets, while underutilization may compromise patient outcomes. In one study focusing on polypharmacy in older adults, healthcare expenses nearly doubled for complex CVD cases, illustrating how challenging resource prioritization can be (Kwak et al., 2022; Duan et al., 2024; Gavina et al., 2024). Moreover, resource distribution often does not adequately account for high-risk groups, as demonstrated by disparities in healthcare utilization across different risk categories (Gavina et al., 2024).

Data Limitations: Accurate adherence to DRG requirements relies on robust data collection and analysis, yet this process is frequently undermined by incomplete or inconsistent records. Inaccuracies in ICD-10 coding and insufficient integration of laboratory or demographic information can distort DRG groupings and lead to misalignments in reimbursement (Dai et al., 2024; GOLDMAN et al., 1989; Liu et al., 2014). Initiatives aimed at improving data quality—such as thorough completion of medical records and the adoption of AI-assisted coding—have shown promise in mitigating these challenges (Goldman et al., 1989; Liu et al., 2014).

In summary, DRG systems can help streamline healthcare expenditures, but applying them effectively in CVD care demands careful adjustments to address the inherent complexities of cardiovascular treatment. Strategies that accommodate patient variability, optimize resource use, and enhance data integrity are crucial for achieving both financial stability and high-quality care.

## 4 Deep learning for clinical pathway optimization

Optimizing clinical pathways for CVD management involves a complex interplay of achieving cost-effectiveness while maintaining or improving patient outcomes. With the increasing adoption of DRG payment systems, there is a pressing need to align healthcare delivery with predefined cost benchmarks. Deep learning has emerged as a transformative tool for addressing these challenges, offering innovative approaches to streamline clinical pathways, predict patient outcomes, and optimize resource utilization.

### 4.1 Goals of optimization

Clinical pathway optimization through deep learning aims to balance cost-efficiency with high-quality, patient-centered care. The key objectives include minimizing unnecessary diagnostics and treatments, shortening hospital stays, adhering to DRG cost benchmarks, enhancing patient satisfaction, improving resource allocation, facilitating early interventions, and enabling real-time decision-making. Each objective is detailed below:

1) Minimizing Unnecessary Diagnostics and Treatments: Deep learning models can help identify low-value or redundant interventions, thereby reducing expenses without undermining diagnostic accuracy or therapeutic efficacy. Under a DRG model, where hospitals receive a fixed payment per case, eliminating such low-value interventions is a critical strategy to manage costs within the predetermined reimbursement threshold and maintain financial viability (Chien et al., 2020). For instance, predictive analytics that evaluate test-ordering patterns can flag superfluous laboratory or imaging requests, curbing overuse and associated costs (Rabbani et al., 2023). Similar frameworks have shown the potential to enhance diagnostic precision while lowering the rate of low-yield tests, thus protecting patients from avoidable procedures and streamlining clinical workflows (Pavuluri et al., 2024; Pomponio et al., 2020).
2) Shortening Hospital Stays Without Compromising Outcomes: Predictive models can project patient recovery patterns, enabling personalized care plans that accelerate recovery while maintaining safety and effectiveness. This capability is particularly vital under DRG payment models, as each day of hospitalization incurs costs, and exceeding the DRG-specified average length of stay (LOS) for a given condition can lead to significant, unreimbursed financial losses for the institution (Kutz et al., 2019; Aragon et al., 2022). Artificial intelligence (AI)-driven systems that integrate electronic health records (EHRs) with machine learning algorithms have demonstrated the capacity to project optimal recovery timelines, thereby reducing hospitalization durations (Ahmed et al., 2020). Additionally, real-time monitoring of patient progress—using data from wearable devices and AI analytics—supports timely treatment adjustments, aiding in earlier discharges and lowering readmission rates (Chen et al., 2023; Javaid et al., 2022).
3) Aligning Costs with DRG Benchmarks: Deep learning tools can pinpoint cost drivers in clinical workflows, recommending modifications that satisfy DRG payment constraints while preserving favorable outcomes (Ravi et al., 2017; Shameer et al., 2018). For example, predictive models can highlight overlapping diagnostics, allowing for targeted cost-cutting measures without compromising quality3. Reinforcement learning algorithms further explore alternative treatment routes to ensure cost-effectiveness, demonstrating how AI can adapt clinical pathways to meet financial targets (Shameer et al., 2018; Shah et al., 2019). Integrating predictive models into everyday practice has yielded more efficient resource allocation strategies, facilitating alignment with DRG benchmarks (Danda and Dileep, 2024).
4) Improving Resource Allocation: Predictive analytics can optimize the distribution of limited, high-cost healthcare resources, such as ICU beds, personnel, and specialized equipment, ensuring maximum utility and cost-effectiveness. Within a DRG framework, the cost of these high-intensity resources must be covered by a single, bundled payment. Therefore, using predictive models to ensure such resources are allocated only to patients who will truly benefit is essential to prevent cost overruns on complex cases (Aragon et al., 2022; King et al., 2022). This allows administrators to proactively redistribute resources and minimize operational bottlenecks (Chen and Asch, 2017). Machine learning systems can also detect inefficiencies across various departments, ensuring fair resource distribution and promoting cost savings (Rajkomar et al., 2019; Talaat, 2024). In DRG-based settings, such methods align hospital resources with cost-effectiveness targets, maintaining high standards of care while controlling expenditures (Li et al., 2023).
5) Facilitating Early Intervention: Deep learning models that incorporate patient vitals, laboratory results, and clinical data can anticipate complications, providing crucial lead time for preventive measures. From a DRG perspective, preventing complications is paramount. A predictable recovery pathway is manageable within a fixed budget; an unexpected complication can trigger a cascade of costly interventions that quickly exceed the DRG reimbursement (Hughes et al., 2018; Mehra et al., 2015). Some algorithms can detect conditions like sepsis up to 48 h before onset, allowing earlier interventions and mitigating severity (Alanazi et al., 2023; Barton et al., 2019). Moreover, explainable AI systems that highlight specific risk factors empower clinicians to respond promptly and effectively, reducing complications and improving patient outcomes (Lundberg et al., 2018).
6) Supporting Real-Time Decision-Making: Integrating deep learning models with live patient data enables immediate adjustments to therapeutic approaches. In the context of DRG-based care, which treats an entire hospitalization as a single paid episode, such real-time adjustments are crucial (Liu J. et al., 2021). Advanced architectures, such as LSTM networks, can process real-time vital sign fluctuations, alerting clinicians to critical changes that warrant rapid intervention. By reducing response times and enhancing situational awareness, such systems contribute to better clinical outcomes and more efficient use of healthcare resources (El-Ganainy et al., 2020; Krittanawong et al., 2019; Sendak et al., 2020).

### 4.2 Techniques and models

Deep learning offers a diverse range of methods tailored to different facets of clinical pathway optimization. These approaches, described below, support both diagnostic and therapeutic decision-making in cardiovascular care while aligning with cost-effectiveness and quality standards.

1) Convolutional Neural Networks (CNNs): CNNs are especially well-suited for image-based analyses, such as evaluating echocardiograms, cardiac MRIs, and CT scans. Their capacity for detecting abnormalities and quantifying disease progression supports more precise diagnostic workflows (Moradi et al., 2024; Jafari et al., 2023). Within a DRG framework, this enhanced diagnostic precision is critical. An accurate, AI-assisted diagnosis helps ensure correct DRG coding from the outset, preventing potential revenue loss from under-coding a complex case (Liu J. et al., 2021; Islam et al., 2021). In cardiac imaging, studies have also reported that CNNs can substantially enhance the early detection of cardiovascular diseases. This allows for the optimization of imaging protocols, which directly reduces costs under a fixed DRG payment by eliminating redundant or low-yield scans that would otherwise erode the hospital’s margin for that episode of care (Li et al., 2023; Liu et al., 2023).

2) RNNs and LSTM: RNNs, and particularly LSTM architectures, excel at processing sequential data, including laboratory values, vital signs, and historical treatment records. LSTMs, for instance, have been shown to predict critical events like heart failure exacerbations or arrhythmias with high accuracy. For example, nested LSTM models have achieved superior prediction accuracy for events such as sudden cardiac death, enabling timely interventions (Wang K. et al., 2024; Rai et al., 2020). CNN-LSTM-SE models further enhance classification tasks like arrhythmia detection by integrating spatial and temporal features, achieving classification accuracies exceeding 97% in certain applications (Nagarajan et al., 2021; Sun et al., 2024). This capability to anticipate and prevent severe adverse events is a primary cost-containment strategy under DRG systems (Wang K. et al., 2024; Sudha and Kumar, 2023). An unforeseen complication can trigger a cascade of intensive, high-cost interventions, pushing the total cost of care far beyond the fixed reimbursement for the patient’s assigned DRG. By enabling proactive intervention, these models help maintain a predictable clinical and financial pathway, thus mitigating the risk of substantial financial losses associated with prolonged hospital stays and unplanned ICU admissions (Hughes et al., 2018; Bruyneel et al., 2025).

3) Reinforcement Learning (RL): Reinforcement learning algorithms are uniquely suited for optimizing clinical pathways within the strict financial constraints of DRG systems. Unlike other models, RL dynamically adapts treatment strategies by learning a policy that maximizes a cumulative reward over a patient’s entire hospital stay (Komorowski et al., 2018; Jayaraman et al., 2024). In a DRG context, this reward function can be explicitly designed to align with financial incentives—for example, by rewarding decisions that lead to successful outcomes while keeping total costs below the fixed DRG payment (Yu et al., 2023). By integrating real-time data from EHRs and wearable devices, RL models can recommend the most cost-effective sequence of interventions (e.g., choosing a less expensive diagnostic test or de-escalating care when a patient is stable), thereby navigating the trade-offs between clinical efficacy and budgetary limits in a data-driven manner (Yu et al., 2023; Yom-Tov et al., 2017).

4) Transformers: Originally developed for natural language processing (NLP), transformers offer a powerful solution for a critical challenge in DRG-based reimbursement: ensuring accurate clinical coding. Under DRG systems, payment is directly determined by the diagnostic and procedural codes (e.g., ICD-10) extracted from clinical documentation. Transformers excel at analyzing unstructured text in EHRs, such as physician notes and discharge summaries, to automatically identify all relevant diagnoses and comorbidities that affect DRG assignment (Denecke et al., 2024). This capability is crucial for preventing under-coding, which can lead to significant revenue loss for healthcare providers. Beyond coding, their ability to process large-scale and multimodal datasets makes them ideal for building more robust predictive models for DRG-related outcomes like cost overruns and readmission risk (Liu J. et al., 2021; Caruso et al., 2024; Ruan et al., 2023). Frameworks like the P-Transformer, which effectively manage data variability in medical tabular data, and models such as MARIA, which handle incomplete data by integrating both structured and unstructured sources, enhance the reliability of these predictions in real-world clinical settings where data is often imperfect (Caruso et al., 2024; Ruan et al., 2023).

5) Graph Neural Networks (GNNs): GNNs are exceptionally well-suited for DRG pathway optimization because they can explicitly model a clinical pathway as a complex graph, where diagnostic tests, treatments, patient characteristics, and outcomes are nodes, and the relationships between them are edges. This structure allows for a holistic analysis of the entire care episode (Islam et al., 2021). For instance, GNNs can process this interconnected data to predict the total cost of a patient’s care pathway with high accuracy (Yang et al., 2024). This predictive capability is a cornerstone of proactive DRG management, enabling hospitals to identify high-cost cases in advance and simulate the financial impact of different treatment choices. Furthermore, by analyzing the graph structure, GNNs can pinpoint which nodes or edges (i.e., specific tests or procedures) are the primary drivers of cost overruns or negative outcomes, thereby providing data-driven insights to refine and standardize clinical pathways to better align with DRG financial targets (Tariq et al., 2023; Gong et al., 2024).

6) Hybrid Models: Hybrid models, which combine multiple deep learning techniques, are essential for creating highly accurate predictive tools for DRG management. Patient data is inherently multimodal, containing static features (e.g., demographics), imaging data (e.g., MRI scans), and time-series data (e.g., ECG signals, lab results over time). No single model architecture can effectively leverage all this information. By integrating different models—such as a CNN for image analysis and an RNN for sequential data—a hybrid model can generate a more robust and accurate risk score for DRG-relevant outcomes (Sadr et al., 2024; Almulihi et al., 2022; Shickel et al., 2023; Fritz et al., 2019; Chen et al., 2024). For example, a CNN-LSTM model can predict a patient’s risk of post-operative complications with greater accuracy than either model alone. This enhanced predictive power is critical for DRG cost control, as it allows for more precise patient stratification and proactive resource allocation, helping to prevent costly deviations from the standard care pathway (Sadr et al., 2024; Almulihi et al., 2022).

7) Federated Learning Models: Federated learning directly addresses a fundamental barrier to creating robust DRG optimization models: data silos and patient privacy. A predictive model for DRG costs or complication risks trained on data from a single hospital may not generalize well to other institutions with different patient populations. Federated learning solves this by enabling multiple hospitals to collaboratively train a single, more powerful and accurate model without sharing sensitive patient data, thus complying with regulations like GDPR and HIPAA (Chaddad et al., 2024; Xu et al., 2024; Abbas et al., 2024). For example, several hospitals in a region could use federated learning to build a shared model that more accurately predicts 30-day readmission risks—a key DRG quality metric. This superior, generalizable model allows all participating institutions to better manage their financial risk and improve care quality under the DRG system (Alvarez-Romero et al., 2022; Sazdov et al., 2023). The key deep learning techniques and models are summarized in Table 1.

**Table 1:** Key deep-learning techniques for optimising clinical pathways under DRG payment models.

Category	Technique/model	Core features and application in DRG-based optimization
Foundational models	CNNs	Analyzes imaging data (e.g., MRI, CT) to improve diagnostic accuracy, supporting correct DRG coding and reducing unnecessary scans.
	RNNs and LSTM	Processes sequential data (e.g., ECGs) to predict adverse events, helping prevent costly complications and prolonged hospital stays.
Advanced optimization models	Reinforcement learning	Dynamically adapts treatment strategies to balance clinical efficacy with DRG cost constraints.
	Transformers	Processes unstructured and multimodal clinical data to improve coding accuracy and build robust risk models.
	GNNs	Models interdependencies within clinical pathways to identify cost drivers and optimize care sequences.
	Hybrid models	Combines the strengths of other models (e.g., CNN + RNN) for more accurate risk prediction with multimodal data.
Collaborative and privacy models	Federated learning	Enables model training across multiple institutions without sharing sensitive patient data, improving model accuracy while ensuring privacy.

CNNs, convolutional neural networks; RNNs, recurrent neural networks; LSTM, long short-term memory; GNNs, graph neural networks; DRG, diagnosis-related group; ECG, electrocardiogram; MRI, magnetic resonance imaging; CT, computed tomography.

**Figure 2:**
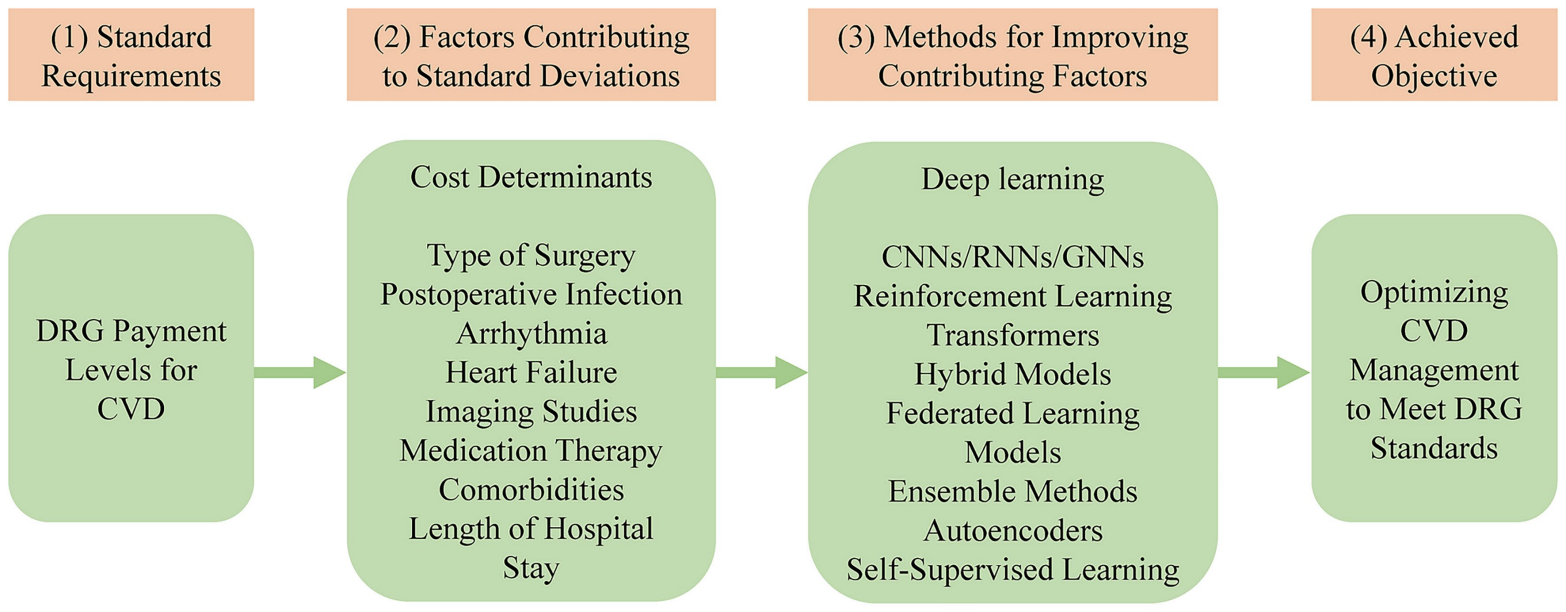
A framework for optimizing CVD management using deep learning to align with DRG standards. DRG, diagnosis-related group; CVD, cardiovascular disease; CNNs, convolutional neural networks; RNNs, recurrent neural networks; GNNs, graph neural networks.

While deep learning techniques provide powerful tools for clinical pathway optimization, their practical implementation in a DRG environment requires careful consideration of their distinct trade-offs. For instance, the need for large, accurately labeled datasets for training models like CNNs translates into a significant data curation cost and effort for hospitals. Similarly, the high computational requirements of advanced methods like transformers can represent a substantial upfront investment in hardware and infrastructure. Even models that offer high robustness, such as hybrid and ensemble approaches, introduce implementation complexity that can strain a hospital’s IT resources and expertise. Therefore, selecting the right technique involves balancing predictive power against these real-world operational and financial constraints. Figure 2 presents a conceptual framework depicting how DRG payment levels for CVD relate to key cost determinants and clinical deviations, the deep learning techniques employed to address these challenges, and the resulting objective of optimizing CVD management to meet DRG requirements.

## 5 Applications of deep learning in DRG-based cardiovascular care

The practical application of deep learning within DRG-based systems offers tangible solutions to long-standing challenges in cardiovascular care, particularly in managing costs and optimizing workflows. The following examples underscore how various techniques can address these challenges, ultimately enhancing both patient outcomes and financial performance under a value-driven reimbursement model.

### 5.1 Predicting intensive monitoring needs

Deep learning models, such as CNNs, can analyze clinical and imaging data to predict which patients are at high risk for post-operative complications like heart failure or arrhythmias. This predictive capability is crucial for proactive resource management within a DRG framework. For instance, studies on hybrid deep learning models for heart disease prediction have reported accuracies as high as 97.17% (Almulihi et al., 2022). By flagging high-risk patients early, these models enable hospitals to optimize the use of high-cost resources like ICU beds. In a DRG model, where the cost of an entire hospital stay—including any necessary ICU care—is bundled into a single fixed payment, this foresight is a critical financial strategy. It helps prevent unforeseen cost overruns and ensures that intensive monitoring is allocated to patients who need it most, thereby aligning clinical needs with budgetary realities (Almulihi et al., 2022; Baccouche et al., 2020).

### 5.2 Identifying high-risk patients

Algorithms like LSTMs are used to stratify patients by risk, which is not merely a clinical exercise but a core component of financial management under DRG systems. For example, by analyzing temporal data such as echocardiographic wall motion, deep learning models can predict the risk of myocardial infarction with high accuracy. To illustrate, one computational analysis demonstrated that an advanced ensemble model could achieve sensitivity and specificity as high as 100 and 99%, respectively. It is important to note, however, that this high performance was achieved through internal validation on a small, public benchmark dataset (the Cleveland dataset, *N* = 303), not on a large, diverse clinical cohort (Midhun et al., 2023). Under a DRG model, accurately identifying patients at high risk for complications is essential because these are the cases most likely to incur costs that exceed the fixed reimbursement. This foresight allows hospitals to implement targeted, cost-effective preventive strategies for these specific patients, rather than applying costly interventions across the board. By doing so, they can efficiently allocate resources to mitigate the risk of financial losses from high-cost outliers, demonstrating the value of AI in aligning patient care with DRG-based financial realities (Midhun et al., 2023; Naidu et al., 2024).

### 5.3 Optimizing post-discharge care

Optimizing post-discharge care is a key strategy for hospitals operating under DRG systems, many of which impose direct financial penalties for high 30-day readmission rates. Machine learning models are powerful tools for designing individualized post-discharge plans that mitigate this financial risk. For example, a large-scale retrospective study analyzing Taiwan’s nationwide health insurance database demonstrated the potential of this approach. The study developed a deep learning model that predicted 30-day readmission for survivors of in-hospital cardiac arrest with an Area Under the ROC Curve (AUROC) of 0.862 (Lu et al., 2021). This enables hospitals to move from a one-size-fits-all approach to a more targeted strategy: accurately stratifying patients at discharge and providing cost-effective follow-up interventions, such as telehealth monitoring, only to those at highest risk. This precision is essential for preventing avoidable readmissions and their associated DRG penalties (Marafino et al., 2021).

### 5.4 Streamlining diagnostic pathways

In a DRG system, the initial diagnosis determines the entire financial and clinical trajectory of a patient’s hospital stay. Deep learning models, particularly CNNs, can streamline this critical first step. By providing a rapid and highly accurate analysis of initial diagnostic images, CNNs help establish the correct diagnosis faster (Ghorbani et al., 2020). This early accuracy is vital for creating a DRG-compliant clinical pathway from the outset, minimizing diagnostic detours and redundant tests that are not separately reimbursed under a bundled payment. This approach not only reduces unnecessary procedures and their associated costs but also ensures that resource allocation and treatment planning are optimized from day one, increasing the likelihood that the total cost of care will remain within the DRG benchmark (Liu J. et al., 2021; Goldsmith et al., 2025).

### 5.5 Improving surgical planning

For high-cost interventions like coronary artery bypass grafting (CABG), surgical planning is a critical control point for DRG management. Machine learning models have enhanced this process by providing robust, data-driven decision support. For example, by integrating patient demographics and imaging data, predictive models can help clinicians choose the most cost-effective revascularization strategy for a specific patient (e.g., CABG vs. PCI), a decision that profoundly impacts the overall cost and DRG assignment (Ninomiya et al., 2023; Cruz et al., 2024). Furthermore, studies evaluating CABG procedures demonstrate that these models improve resource allocation by predicting the likely complexity and needs of the surgery in advance. This foresight ensures that resource-intensive interventions are justified and planned efficiently, aligning the clinical plan with the financial realities of a fixed DRG payment from the very beginning (Cruz et al., 2024; Verma et al., 2022).

### 5.6 Validating workflow efficiencies in acute care

For many patients, the clinical pathway and its associated costs begin in the emergency department (ED), making workflow efficiency in this setting critical for DRG management. Real-world implementations have shown that AI tools can significantly streamline these acute care pathways. For instance, studies emphasize that CNN-based diagnostic systems for acute coronary syndrome (ACS) in the ED not only improve diagnostic accuracy but also accelerate the entire care process. By enabling faster and more accurate triage—such as by rapidly identifying ST-elevation myocardial infarction (STEMI) from an ECG—these systems reduce time-to-treatment. Under a DRG model, this acceleration is crucial for minimizing the length of stay and preventing costly delays or diagnostic detours that are not reimbursed. This demonstrates how validating and implementing AI-driven workflow efficiencies directly contributes to better financial and clinical outcomes within a bundled payment system (Liu W. C. et al., 2021; Demandt et al., 2025).

## 6 Integration into clinical workflows

For deep learning–driven optimizations to generate tangible value within a DRG-based payment system, they must be seamlessly incorporated into established clinical processes. Such integration is not merely a technical challenge but a strategic imperative, ensuring that AI tools directly support clinical and administrative staff in meeting DRG-defined targets for cost and quality. Key considerations, which are summarized in Table 2, include:

**Table 2:** A practical implementation framework for integrating deep learning tools into DRG-based workflows.

Phase	Key steps and considerations
Problem and scope definition	Identify a high-cost or high-variance DRG to target. Define clear success metrics (e.g., reduce LOS, lower readmission penalties).
Data and infrastructure	Assess quality, accessibility, and completeness of required data (EHR, imaging, etc.). Evaluate existing IT infrastructure and computational resources.
Model development and validation	Select an appropriate model based on the problem and data type. Plan for rigorous internal and, crucially, external validation. Conduct fairness audits to identify and mitigate potential algorithmic bias.
Workflow integration	Co-design user interfaces with frontline clinicians to ensure usability. Provide comprehensive staff training on how to interpret and act on model outputs. Ensure seamless integration into the EHR to avoid disrupting clinical routines.
Governance and monitoring	Ensure full compliance with regulatory (e.g., HIPAA, GDPR) and ethical standards. Continuously track key clinical outcomes and financial metrics (e.g., ROI, cost-per-case) post-implementation.

DRG, diagnosis-related group; LOS, length of stay; EHR, electronic health record; HIPAA, health insurance portability and accountability act; GDPR, general data protection regulation; ROI, return on investment.

### 6.1 Real-time decision support

Embedding predictive models into EHR systems is crucial for managing the entire episode of care under a single DRG payment. These tools enable clinicians to act on real-time, actionable insights at the point of care (Adams et al., 2022). For instance, an RNN integrated into an EHR can identify elevated readmission risks, prompting timely interventions that can prevent a costly and often uncompensated new admission. Studies have demonstrated that AI tools embedded in EHRs can reduce adverse events significantly through proactive alerts and tailored recommendations (Shetty et al., 2022; Xiao et al., 2018; Choi et al., 2024; Zhao et al., 2021; Wu et al., 2021).

Similarly, advanced decision support systems that integrate risk calculators for conditions like sepsis or acute kidney injury lead to measurable reductions in hospital-acquired complications (Zhao et al., 2021; Wu et al., 2021). In a DRG environment, preventing such complications is a primary financial strategy, as they can trigger a cascade of high-cost services that are not adequately covered by the initial bundled payment, thereby improving both clinical outcomes and resource efficiency.

### 6.2 Interdisciplinary collaboration for DRG-focused implementation

Successful AI implementation within a DRG framework mandates a broad interdisciplinary collaboration that extends beyond the traditional team of data scientists and clinicians. Crucially, this team must include hospital administrators, financial analysts, and DRG coding specialists. While clinicians provide essential domain expertise to ensure model outputs are clinically relevant, it is the administrators and financial experts who must align these innovations with the specific financial targets and operational constraints imposed by the DRG system (Adams et al., 2022; Henry et al., 2022). Studies have demonstrated that this kind of collaborative design enhances not only user satisfaction but also the successful adoption of tools aimed at improving DRG-related outcomes (Ahmad et al., 2023; Mittermaier et al., 2023; Elgin and Elgin, 2024). By fostering a culture where clinical, technical, and financial stakeholders work together, organizations can ensure that AI solutions are built not just to be clinically effective, but also to be economically viable and sustainable under a bundled payment model (Stogiannos et al., 2024; Maleki Varnosfaderani and Forouzanfar, 2024).

### 6.3 Ensuring workflow compatibility

To be successful, AI tools must integrate seamlessly into existing clinical workflows; in a DRG environment, any friction or disruption translates directly into unreimbursed labor costs and potential delays that can extend the length of stay. Prospective studies of the TREWS sepsis early-warning system show that embedding an ML model directly into the EHR—and co-designing interfaces with frontline clinicians—can cut time-to-treatment and reduce LOS without adding workflow burden (Henry et al., 2022).

Simplified interfaces and clear data visualizations are therefore not just about user satisfaction, but are critical for minimizing the cognitive and time burden on clinicians, whose time is a core resource that must be managed within a bundled payment. Provider-adoption analyses of the same TREWS system confirm that intuitive, human-centred design is a key driver of sustained use at the bedside (Adams et al., 2022; Choi et al., 2024).

Similarly, a readmission-risk tool integrated into a multisite EHR reduced 30-day rehospitalisations by targeting follow-up resources only to high-risk patients, demonstrating the financial value of seamless workflow integration under DRG penalties (Marafino et al., 2021). Workflow compatibility also requires tailoring systems to the specific needs of high-stakes departments, such as critical care units. Embedding decision support that respects the rapid-response culture of ICUs—rather than adding extra clicks—helps optimise the most resource-intensive portions of a DRG episode, thereby maximising both clinical and financial efficiency (Henry et al., 2022).

### 6.4 Regulatory and ethical considerations in DRG optimization

The integration of AI into DRG-based workflows necessitates strict adherence to regulatory and ethical standards, particularly concerning data privacy and algorithmic fairness. Ensuring patient data privacy under regulations like GDPR and HIPAA is paramount, especially as creating robust DRG prediction models often requires diverse, multi-institutional data. Federated learning models offer a critical technical solution to this challenge, enabling collaborative model training without centralizing sensitive patient data (Rieke et al., 2020).

Perhaps the most significant ethical challenge is the risk that AI models, when optimized for cost-efficiency under DRG constraints, may perpetuate or even amplify health disparities (Obermeyer et al., 2019). If historical data reflects that certain vulnerable populations received less intensive care, an AI model might learn to recommend lower-cost (and potentially suboptimal) clinical pathways for these groups, creating a system of automated bias. Therefore, minimizing bias is not just a technical goal but an ethical imperative to ensure equitable resource allocation under DRG’s financial pressures. Institutions must adopt rigorous fairness audits and transparent frameworks to ensure that AI-driven optimizations genuinely improve value for all patient demographics, not just reduce costs for the system (Rajkomar et al., 2018).

### 6.5 Measuring impact on operational and financial efficiency

To justify AI investment, continuous evaluation of both clinical and operational outcomes is essential within a DRG framework. Although isolating direct cost savings is challenging, robust indicators—such as reduced length of stay, earlier antibiotic administration, and more accurate early CMI forecasts for staffing and theatre allocation—directly reflect AI-driven gains in DRG performance (Liu J. et al., 2021; Adams et al., 2022).

### 6.6 Overcoming barriers to DRG-focused AI implementation

Overcoming barriers to AI adoption is particularly challenging in a DRG context, as it requires balancing clinical needs with strict financial constraints. Key obstacles include developing models that are robust enough to guide real-world financial decisions, establishing clear institutional policies on how to act on AI-driven cost-saving recommendations, and accurately measuring the impact on DRG-specific metrics.

Successful implementation hinges on strategic stakeholder engagement, bringing together clinical teams with hospital administrators, financial analysts, and data scientists to align on shared DRG-related goals (Wolff et al., 2021). Phased rollouts and pilot projects are essential, not just to test technical feasibility, but to validate the economic return on investment (ROI) for specific DRG cohorts. By proactively addressing these unique challenges, AI systems can be integrated not merely as clinical aids, but as essential tools for achieving financially sustainable, high-quality care in a value-based payment environment (Bizzo et al., 2023; Smith et al., 2021).

## 7 Discussion

The convergence of deep learning, CVD management, and DRG payment systems offers transformative possibilities for enhancing healthcare delivery. This section synthesizes key insights and challenges from the preceding analysis, providing a critical perspective on the current state of these technologies and potential avenues for further research.

### 7.1 Deep learning in CVD management: bridging gaps in care

Deep learning models, such as CNNs and RNNs, have shown significant potential in diagnosing and managing CVDs. For example, studies demonstrate that CNNs can improve myocardial infarction detection by analyzing imaging data, reducing unnecessary diagnostic tests, and enabling timely interventions. RL approaches, particularly in optimizing dynamic treatment strategies, have demonstrated promising results. An RL-based system for coronary heart disease treatment reduced estimated in-hospital mortality by 3.13%, showcasing its potential to improve patient outcomes while aligning with DRG payment benchmarks (Talaat, 2024; Guo et al., 2022; Drudi et al., 2024).

Despite these advancements, challenges remain in scaling these technologies. Variability in data quality and limited interpretability of AI models can hinder adoption. Additionally, integrating AI into existing clinical workflows without causing disruptions requires careful planning and stakeholder collaboration. Multidisciplinary approaches that combine technological innovation with clinical and operational expertise are essential. Furthermore, patient-centric strategies must prioritize ethical considerations and equitable access to ensure that technological solutions align with broader healthcare goals. By addressing these barriers and leveraging the unique capabilities of deep learning, healthcare systems can achieve enhanced clinical outcomes and more efficient cost management under DRG frameworks.

### 7.2 Challenges in aligning deep learning with DRG systems

DRG-based payment mechanisms emphasize cost containment while maintaining high-quality care, posing unique challenges in CVD management. Key cost drivers—such as high-cost interventions, longer hospital stays, and advanced diagnostic procedures—can be mitigated by deep learning tools that identify at-risk patients earlier, forecast complications, and recommend efficient resource allocation. However, four major obstacles persist:

1) Standardization of Care: Variability in patient responses remains a significant barrier to the implementation of standardized care pathways under DRG reimbursement structures. While recent studies highlight the potential of advanced deep learning models to integrate multimodal data (e.g., EHRs, imaging) for improving comorbidity predictions, challenges remain in generalizing these methods across diverse patient populations. For instance, demonstrates the utility of convolutional neural networks in mortality prediction, but questions about model interpretability and scalability in resource-limited settings persist (Pyrros et al., 2022). Furthermore, emphasizes the integration of structured and unstructured EHR data to address demographic variability, enhancing the adaptability of predictive models in diverse clinical settings (Nasarudin et al., 2024). Additionally, the fairness-aware frameworks proposed address systemic biases but require further validation in real-world settings with heterogeneous data (Wang Y. et al., 2024). Future research should explore robust mechanisms for ensuring both model accuracy and equity, particularly in underrepresented patient groups.
2) Data Quality and Accessibility: Inconsistent or incomplete datasets significantly impede the training and validation of predictive models. The literature highlights that high-dimensional and multimodal data, including EHRs, often suffer from issues such as missing values, poor data quality, and a lack of standardization (He et al., 2023; Luo et al., 2022). Advanced data integration approaches, such as combining structured and unstructured data or generating synthetic data using advanced generative models (e.g., diffusion models), have been proposed to address these challenges (Nasarudin et al., 2024; He et al., 2023). Furthermore, causal representation learning frameworks have demonstrated potential for mitigating biases and improving data stability across diverse clinical environments, thereby enhancing the reliability of predictive models (Luo et al., 2022). Future research should prioritize the development of robust methodologies to harmonize and enhance EHR data for improved model performance and generalizability.
3) Financial Constraints: The high initial investment required for deploying deep learning systems often poses a significant barrier, particularly in resource-limited settings. According to, financial constraints are exacerbated by a lack of clear return on investment (ROI) in the early phases of implementation, as well as skepticism regarding cost-effectiveness in the healthcare domain (Chomutare et al., 2022). Similarly, highlights that successful AI adoption in healthcare often depends on well-structured funding models, including government subsidies, public-private partnerships, and cost-sharing mechanisms, to offset the financial burden (Joshi et al., 2022). Future efforts should focus on creating sustainable financing strategies that prioritize long-term benefits while addressing immediate cost barriers, ensuring equitable access to AI-driven solutions in healthcare.
4) Operational Bottlenecks: The implementation of AI-driven tools in healthcare management frequently reveals underlying inefficiencies in hospital operations. According to, these inefficiencies are often related to resource allocation, workflow integration, and administrative processes. Addressing these issues requires a dual focus on operational optimization and the adoption of AI. For instance, AI-powered systems have demonstrated potential in streamlining resource allocation and improving patient flow, yet their success heavily depends on aligning these technologies with existing hospital infrastructures. Furthermore, proactive measures to identify and resolve these inefficiencies during the implementation phase can significantly enhance the overall effectiveness of AI-driven solutions. This integrated approach ensures smoother transitions, improved healthcare delivery, and sustainable outcomes (Dubey et al., 2023).

### 7.3 Integration into clinical workflows

Effective integration of deep learning into clinical workflows requires addressing both technical and socio-technical challenges. Real-time decision support models embedded within EHR systems can streamline diagnostics and reduce clinician workload. However, as noted, their success depends on usability, trust, and alignment with clinical workflows. For instance, studies have shown that AI tools designed with clinician input can enhance adoption by addressing user needs and reducing the cognitive burden of manual data input (Rajashekar et al., 2024). Furthermore, emphasizes the importance of transparency and ongoing education in maintaining clinician trust and ensuring seamless implementation (Finkelstein et al., 2024). Beyond technical performance, highlights the need for interdisciplinary collaboration to adapt AI systems to the dynamic and context-specific needs of clinical environments (Wang et al., 2023). This ensures that the full potential of AI is realized, transforming systemic care delivery by fostering better decision-making, improved efficiency, and enhanced patient outcomes.

### 7.4 Addressing real-world evidence gaps

Despite progress in the application of deep learning, significant gaps in real-world evidence persist, hindering its broad clinical adoption. A primary challenge stems from the current literature’s reliance on retrospective, single-center studies that often lack external validation, limiting their generalizability across different patient populations and healthcare settings (Srivastava et al., 2023; Lee et al., 2022; Cai et al., 2024). To build trust and ensure clinical applicability, there is a critical need for more robust, prospective, multi-center validation studies that evaluate these AI models in diverse, real-world environments (Lee et al., 2022). Moreover, a significant evidence gap exists in the quantification of financial outcomes. While many models claim the potential for cost savings, there is a scarcity of research that rigorously measures the return on investment (ROI) of these tools in terms of direct cost reductions or improved DRG margins (Cho et al., 2023). Bridging these evidence gaps through high-quality validation studies and transparent financial analyses is essential for moving AI-driven solutions from research to routine clinical practice.

### 7.5 Broader impacts and ethical dimensions

1) Equity in AI Deployment: AI deployment in low-resource settings is hindered by prohibitive hardware costs, inadequate data for model training, and a shortage of technical expertise. Moreover, the diversity of global DRG systems poses an equity challenge. AI models developed for complex North American or European systems may perform poorly in regions with different structures. Applying these models without modification risks creating “one-size-fits-all” solutions that widen global health disparities. Therefore, to ensure fairness, AI frameworks must be adaptable to local contexts.
2) Transparency and Trust: Clinicians and patients must trust AI recommendations, underscoring the importance of interpretable models. Initiatives to demystify AI decision-making processes, often through the application of Explainable AI (XAI) techniques, can foster wider acceptance.
3) Continuous Learning: AI systems must adapt to new evidence and shifting clinical guidelines. Dynamic models capable of continuous updates ensure relevance while avoiding obsolescence.
4) Limitations: This review provides a robust synthesis of existing evidence but has limitations. It predominantly relies on secondary data sources and may not fully capture unpublished or emerging innovations. Additionally, while this paper explores scalable solutions, further work is needed to address specific challenges in underrepresented healthcare environments.

### 7.6 Future directions and research priorities

#### 7.6.1 Technical and model development

1) Dynamic Model Adaptation: Continuous learning algorithms that adapt to evolving clinical guidelines and patient populations will enhance the relevance and accuracy of AI tools.
2) Multi-Modal Data Integration: Combining genomic, imaging, and patient-reported outcomes with EHR data can provide a more holistic view of patient health, enabling personalized care pathways.
3) Addressing Data Dependency: Researchers must develop methods to reduce reliance on large-scale, labeled datasets, such as self-supervised learning or synthetic data generation.
4) Advancing Toward Causal Inference: A critical future direction is to move beyond correlation-based prediction to causal inference. Developing models that can help explain why a particular treatment pathway is likely to be effective for a patient—not just predicting that it will be—will be essential for creating more robust, trustworthy, and clinically actionable AI systems.

#### 7.6.2 Clinical validation and implementation science

1) Outcome Monitoring: Longitudinal studies are necessary to validate and refine these technologies by tracking specific DRG-relevant outcomes (e.g., length of stay, 30-day readmission rates) alongside financial metrics such as total cost per episode versus the fixed DRG reimbursement.
2) Resource Optimization Studies: Research focused on optimizing resource allocation using AI insights can help healthcare systems achieve cost-efficiency while maintaining high care standards.

#### 7.6.3 Accessibility, governance, and ethics

1) Scalability and Accessibility: Expanding access requires cost-effective deployment strategies. Research should prioritize lightweight, computationally efficient models for low-resource settings, alongside modular AI frameworks that can be adapted to diverse global DRG systems. Leveraging cloud platforms and techniques like transfer learning will be crucial to enhance scalability and overcome data limitations.
2) Collaborative Networks: Establishing global networks for AI development and deployment can facilitate knowledge sharing and accelerate advancements. Open-source platforms and cross-border collaborations will be instrumental in scaling innovations.
3) Ethical Considerations: Tackling potential biases and ensuring fairness in model recommendations will be crucial in widespread AI adoption.

## 8 Conclusion

Deep learning offers significant opportunities to optimize clinical pathways for CVD management, aligning with DRG payment systems to balance cost control and quality care. While challenges in data quality, workflow integration, and ethical considerations remain, collaborative efforts among key stakeholders—including clinicians, data scientists, hospital administrators, and policymakers—can overcome these barriers. By focusing on scalability, interoperability, and real-world validation, deep learning-driven solutions can pave the way for a more equitable and efficient healthcare system.

## References

[ref1] Abbas S. R. Abbas Z. Zahir A. Lee S. W. (2024). Federated learning in smart healthcare: a comprehensive review on privacy, security, and predictive analytics with IoT integration. Healthcare 12:587. doi: 10.3390/healthcare12242587, PMID: 39766014 PMC11728217

[ref2] Adams R. Henry K. E. Sridharan A. Soleimani H. Zhan A. Rawat N.. (2022). Prospective, multi-site study of patient outcomes after implementation of the TREWS machine learning-based early warning system for sepsis. Nat. Med. 28, 1455–1460. doi: 10.1038/s41591-022-01894-0, PMID: 35864252

[ref3] Adedinsewo D. Carter R. E. Attia Z. Johnson P. Kashou A. H. Dugan J. L.. (2020). Artificial intelligence-enabled ECG algorithm to identify patients with left ventricular systolic dysfunction presenting to the emergency department with dyspnea. Circ. Arrhythm. Electrophysiol. 13:e008437. doi: 10.1161/CIRCEP.120.008437, PMID: 32986471

[ref4] Ahmad N. Du S. Ahmed F. ul Amin N. Yi X. (2023). Healthcare professionals satisfaction and AI-based clinical decision support system in public sector hospitals during health crises: a cross-sectional study. Inf. Technol. Manag. 26, 205–217. doi: 10.1007/s10799-023-00407-w

[ref5] Ahmed Z. Mohamed K. Zeeshan S. Dong X. (2020). Artificial intelligence with multi-functional machine learning platform development for better healthcare and precision medicine. Database 2020:10. doi: 10.1093/database/baaa010, PMID: 32185396 PMC7078068

[ref6] Alanazi A. Aldakhil L. Aldhoayan M. Aldosari B. (2023). Machine learning for early prediction of sepsis in intensive care unit (ICU) patients. Medicina 59:276. doi: 10.3390/medicina59071276, PMID: 37512087 PMC10385427

[ref7] Almulihi A. Saleh H. Hussien A. M. Mostafa S. El-Sappagh S. Alnowaiser K.. (2022). Ensemble learning based on hybrid deep learning model for heart disease early prediction. Diagnostics 12:215. doi: 10.3390/diagnostics12123215, PMID: 36553222 PMC9777370

[ref8] Alvarez-Romero C. Martinez-Garcia A. Ternero Vega J. Díaz-Jimènez P. Jimènez-Juan C. Nieto-Martín M. D.. (2022). Predicting 30-day readmission risk for patients with chronic obstructive pulmonary disease through a federated machine learning architecture on findable, accessible, interoperable, and reusable (FAIR) data: development and validation study. JMIR Med. Inform. 10:e35307. doi: 10.2196/35307, PMID: 35653170 PMC9204581

[ref9] Aragon M. J. Chalkley M. Kreif N. (2022). The long-run effects of diagnosis related group payment on hospital lengths of stay in a publicly funded health care system: evidence from 15 years of micro data. Health Econ. 31, 956–972. doi: 10.1002/hec.4479, PMID: 35238106 PMC9314794

[ref10] Attia Z. I. Kapa S. Lopez-Jimenez F. McKie P. M. Ladewig D. J. Satam G.. (2019). Screening for cardiac contractile dysfunction using an artificial intelligence-enabled electrocardiogram. Nat. Med. 25, 70–74. doi: 10.1038/s41591-018-0240-2, PMID: 30617318

[ref11] Baccouche A. Garcia-Zapirain B. Castillo Olea C. Elmaghraby A. (2020). Ensemble deep learning models for heart disease classification: a case study from Mexico. Information 11:207. doi: 10.3390/info11040207

[ref12] Baral S. Alsadoon A. Prasad P. W. C. Al Aloussi S. Alsadoon O. H. (2021). A novel solution of using deep learning for early prediction cardiac arrest in Sepsis patient: enhanced bidirectional long short-term memory (LSTM). Multimed. Tools Appl. 80, 32639–32664. doi: 10.1007/s11042-021-11176-5

[ref13] Barton C. Chettipally U. Zhou Y. Jiang Z. Lynn-Palevsky A. le S.. (2019). Evaluation of a machine learning algorithm for up to 48-hour advance prediction of sepsis using six vital signs. Comput. Biol. Med. 109, 79–84. doi: 10.1016/j.compbiomed.2019.04.027, PMID: 31035074 PMC6556419

[ref14] Bates D. W. Saria S. Ohno-Machado L. Shah A. Escobar G. (2014). Big data in health care: using analytics to identify and manage high-risk and high-cost patients. Health Aff. 33, 1123–1131. doi: 10.1377/hlthaff.2014.0041, PMID: 25006137

[ref15] Benjamin E. J. Muntner P. Alonso A. Bittencourt M. S. Callaway C. W. Carson A. P.. (2019). Heart disease and stroke statistics-2019 update: a report from the American Heart Association. Circulation 139, e56–e528. doi: 10.1161/CIR.0000000000000659, PMID: 30700139

[ref16] Bertsimas D. Orfanoudaki A. Weiner R. B. (2020). Personalized treatment for coronary artery disease patients: a machine learning approach. Health Care Manag. Sci. 23, 482–506. doi: 10.1007/s10729-020-09522-4, PMID: 33040231

[ref17] Bizzo B. C. Dasegowda G. Bridge C. Miller B. Hillis J. M. Kalra M. K.. (2023). Addressing the challenges of implementing artificial intelligence tools in clinical practice: principles from experience. J. Am. Coll. Radiol. 20, 352–360. doi: 10.1016/j.jacr.2023.01.002, PMID: 36922109

[ref18] Bjerken L. V. Ronborg S. N. Jensen M. T. Orting S. N. Nielsen O. W. (2023). Artificial intelligence enabled ECG screening for left ventricular systolic dysfunction: a systematic review. Heart Fail. Rev. 28, 419–430. doi: 10.1007/s10741-022-10283-1, PMID: 36344908 PMC9640840

[ref19] Bruyneel A. den Bulcke J. V. Leclercq P. Pirson M. (2025). Frequency, financial impact, and factors associated with cost outliers in intensive care units: a cohort study in Belgium. Crit. Care Sci. 37:e20250207. doi: 10.62675/2965-2774.20250207, PMID: 39879435 PMC11805458

[ref20] Busse R. Geissler A. Aaviksoo A. Cots F. Häkkinen U. Kobel C.. (2013). Diagnosis related groups in Europe: moving towards transparency, efficiency, and quality in hospitals? BMJ 346, 1–7. doi: 10.1136/bmj.f3197 23747967

[ref21] Cai Y. Cai Y. Q. Tang L. Y. Wang Y. H. Gong M. Jing T. C.. (2024). Artificial intelligence in the risk prediction models of cardiovascular disease and development of an independent validation screening tool: a systematic review. BMC Med. 22:56. doi: 10.1186/s12916-024-03273-7, PMID: 38317226 PMC10845808

[ref22] Caruso C. M. Soda P. Guarrasi V. (2024). MARIA: a multimodal transformer model for incomplete healthcare data. Arxiv 2412, 1–45. doi: 10.1016/j.compbiomed.2025.110843 40784080

[ref23] Centonze M. Steidler S. Casagranda G. Alfonsi U. Spagnolli F. Rozzanigo U.. (2020). Cardiac-CT and cardiac-MR cost-effectiveness: a literature review. Radiol. Med. 125, 1200–1207. doi: 10.1007/s11547-020-01290-z, PMID: 32970273

[ref24] Chaddad A. Wu Y. Desrosiers C. (2024). Federated learning for healthcare applications. IEEE Internet Things J. 11, 7339–7358. doi: 10.1109/JIOT.2023.3325822

[ref25] Chen J. H. Asch S. M. (2017). Machine learning and prediction in medicine-beyond the peak of inflated expectations. N. Engl. J. Med. 376, 2507–2509. doi: 10.1056/NEJMp1702071, PMID: 28657867 PMC5953825

[ref26] Chen E. Prakash S. Janapa Reddi V. Kim D. Rajpurkar P. (2023). A framework for integrating artificial intelligence for clinical care with continuous therapeutic monitoring. Nat. Biomed. Eng. 9, 445–454. doi: 10.1038/s41551-023-01115-0, PMID: 37932379

[ref27] Chen X. Wang X. Zhang K. Fung K. M. Thai T. C. Moore K.. (2022). Recent advances and clinical applications of deep learning in medical image analysis. Med. Image Anal. 79:102444. doi: 10.1016/j.media.2022.102444, PMID: 35472844 PMC9156578

[ref28] Chen J. Wen Y. Pokojovy M. Tseng T. L. McCaffrey P. Vo A.. (2024). Multi-modal learning for inpatient length of stay prediction. Comput. Biol. Med. 171:108121. doi: 10.1016/j.compbiomed.2024.108121, PMID: 38382388

[ref29] Chi C. Y. Ao S. Winkler A. Fu K. C. Xu J. Ho Y. L.. (2021). Predicting the mortality and readmission of in-hospital cardiac arrest patients with electronic health records: a machine learning approach. J. Med. Internet Res. 23:e27798. doi: 10.2196/27798, PMID: 34515639 PMC8477292

[ref30] Chien L. C. Chou Y. J. Huang Y. C. Shen Y. J. Huang N. (2020). Reducing low value services in surgical inpatients in Taiwan: does diagnosis-related group payment work? Health Policy 124, 89–96. doi: 10.1016/j.healthpol.2019.10.005, PMID: 31699446

[ref31] Cho K. J. Kim J. S. Lee D. H. Lee S. M. Song M. J. Lim S. Y.. (2023). Prospective, multicenter validation of the deep learning-based cardiac arrest risk management system for predicting in-hospital cardiac arrest or unplanned intensive care unit transfer in patients admitted to general wards. Crit. Care 27:346. doi: 10.1186/s13054-023-04609-0, PMID: 37670324 PMC10481524

[ref32] Choi A. Lee K. Hyun H. Kim K. J. Ahn B. Lee K. H.. (2024). A novel deep learning algorithm for real-time prediction of clinical deterioration in the emergency department for a multimodal clinical decision support system. Sci. Rep. 14:30116. doi: 10.1038/s41598-024-80268-7, PMID: 39627310 PMC11615388

[ref33] Chomutare T. Tejedor M. Svenning T. O. Marco-Ruiz L. Tayefi M. Lind K.. (2022). Artificial intelligence implementation in healthcare: a theory-based scoping review of barriers and facilitators. Int. J. Environ. Res. Public Health 19:359. doi: 10.3390/ijerph192316359, PMID: 36498432 PMC9738234

[ref34] Chu J. Dong W. Huang Z. (2020). Endpoint prediction of heart failure using electronic health records. J. Biomed. Inform. 109:103518. doi: 10.1016/j.jbi.2020.103518, PMID: 32721582

[ref35] Cinar A. Tuncer S. A. (2021). Classification of normal sinus rhythm, abnormal arrhythmia and congestive heart failure ECG signals using LSTM and hybrid CNN-SVM deep neural networks. Comput. Methods Biomech. Biomed. Engin. 24, 203–214. doi: 10.1080/10255842.2020.1821192, PMID: 32955928

[ref36] Cruz E. O. Sakowitz S. Mallick S. le N. Chervu N. Bakhtiyar S. S.. (2024). Machine learning prediction of hospitalization costs for coronary artery bypass grafting operations. Surgery 176, 282–288. doi: 10.1016/j.surg.2024.03.051, PMID: 38760232

[ref37] Dai H. J. Wang C. K. Chen C. C. Liou C.-S. Lu A.-T. Lai C.-H.. (2024). Evaluating a natural language processing-driven, AI-assisted international classification of diseases, 10th revision, clinical modification, coding system for diagnosis related groups in a real hospital environment: algorithm development and validation study. J. Med. Internet Res. 26:e58278. doi: 10.2196/58278, PMID: 39302714 PMC11452756

[ref38] Damluji A. A. van Diepen S. Katz J. N. Menon V. Tamis-Holland J. E. Bakitas M.. (2021). Mechanical complications of acute myocardial infarction: a scientific statement from the American Heart Association. Circulation 144, e16–e35. doi: 10.1161/CIR.0000000000000985, PMID: 34126755 PMC9364424

[ref39] Danda R. R. Dileep V. (2024). Leveraging AI and machine learning for enhanced preventive care and chronic disease management in health insurance plans. Front. Health Inform. 13, 6878–6891.

[ref40] Demandt J. P. A. Mast T. P. van Beek K. A. J. Koks A. Bastiaansen M. C. V. Tonino P. A. L.. (2025). Towards prehospital risk stratification using deep learning for ECG interpretation in suspected acute coronary syndrome. BMJ Health Care Inform. 32:292. doi: 10.1136/bmjhci-2024-101292, PMID: 40480678 PMC12161418

[ref41] Denecke K. May R. Rivera-Romero O. (2024). Transformer models in healthcare: a survey and thematic analysis of potentials, shortcomings and risks. J. Med. Syst. 48:23. doi: 10.1007/s10916-024-02043-5, PMID: 38367119 PMC10874304

[ref42] Doenst T. Haverich A. Serruys P. Bonow R. O. Kappetein P. Falk V.. (2019). PCI and CABG for treating stable coronary artery disease: JACC review topic of the week. J. Am. Coll. Cardiol. 73, 964–976. 30819365 10.1016/j.jacc.2018.11.053

[ref43] Drudi C. Fechner M. Mollura M. Pace A. Rätsch G. Barbieri R. Reinforcement learning for heart failure treatment optimization in the intensive care unit 2024 46th Annual International Conference of the IEEE Engineering in Medicine and Biology Society (EMBC) (2024). 10.1109/EMBC53108.2024.10781564 40039224

[ref44] Duan J. Jiao F. Xi J. Zhang Q. (2024). Based on knowledge capital value for disease cost accounting of diagnosis related groups. Front. Public Health 12:1269704. doi: 10.3389/fpubh.2024.1269704, PMID: 38915748 PMC11194358

[ref45] Dubey K. Bhowmik M. Pawar A. Patil M. K. Deshpande P. A. Khartad S. S.. Enhancing operational efficiency in healthcare with AI-powered management. 2023 international conference on artificial intelligence for innovations in healthcare industries (ICAIIHI); (2023). p. 1–7.

[ref46] El-Chami M. F. Sawaya F. J. Kilgo P. Stein W. Halkos M. Thourani V.. (2012). Ventricular arrhythmia after cardiac surgery: incidence, predictors, and outcomes. J. Am. Coll. Cardiol. 60, 2664–2671. doi: 10.1016/j.jacc.2012.08.1011, PMID: 23177295

[ref47] El-Ganainy N. O. Balasingham I. Halvorsen P. S. Rosseland L. A. (2020). A new real time clinical decision support system using machine learning for critical care units. IEEE Access 8, 185676–185687. doi: 10.1109/ACCESS.2020.3030031

[ref48] Elgin C. Y. Elgin C. (2024). Ethical implications of AI-driven clinical decision support systems on healthcare resource allocation: a qualitative study of healthcare professionals' perspectives. BMC Med. Ethics 25:148. doi: 10.1186/s12910-024-01151-8, PMID: 39707327 PMC11662436

[ref49] Esteva A. Robicquet A. Ramsundar B. Kuleshov V. DePristo M. Chou K.. (2019). A guide to deep learning in healthcare. Nat. Med. 25, 24–29. doi: 10.1038/s41591-018-0316-z, PMID: 30617335

[ref50] Figtree G. A. Vernon S. T. Harmer J. A. Gray M. P. Arnott C. Bachour E.. (2023). Clinical pathway for coronary atherosclerosis in patients without conventional modifiable risk factors: JACC state-of-the-art review. J. Am. Coll. Cardiol. 82, 1343–1359. doi: 10.1016/j.jacc.2023.06.045, PMID: 37730292 PMC10522922

[ref51] Finkelstein J. Gabriel A. Schmer S. Truong T. T. Dunn A. (2024). Identifying facilitators and barriers to implementation of AI-assisted clinical decision support in an electronic health record system. J. Med. Syst. 48:89. doi: 10.1007/s10916-024-02104-9, PMID: 39292314 PMC11410896

[ref52] Fritz B. A. Cui Z. Zhang M. He Y. Chen Y. Kronzer A.. (2019). Deep-learning model for predicting 30-day postoperative mortality. Br. J. Anaesth. 123, 688–695. doi: 10.1016/j.bja.2019.07.025, PMID: 31558311 PMC6993109

[ref53] Gavina C. Borges A. Afonso-Silva M. Fortuna I. Canelas-Pais M. Amaral R.. (2024). Patients' health care resources utilization and costs estimation across cardiovascular risk categories: insights from the LATINO study. Health Econ. Rev. 14:73. doi: 10.1186/s13561-024-00550-2, PMID: 39264520 PMC11395856

[ref54] Gholami S. S. Azar F. E. F. Rezapour A. Tajdini M. (2019). Cost-effectiveness of coronary artery bypass graft and percutaneous coronary intervention compared to medical therapy in patients with coronary artery disease: a systematic review. Heart Fail. Rev. 24, 967–975. doi: 10.1007/s10741-019-09811-3, PMID: 31179517

[ref55] Ghorbani A. Ouyang D. Abid A. He B. Chen J. H. Harrington R. A.. (2020). Deep learning interpretation of echocardiograms. NPJ Digit. Med. 3:10. doi: 10.1038/s41746-019-0216-8, PMID: 31993508 PMC6981156

[ref56] Goldfield N. (2010). The evolution of diagnosis-related groups (DRGs): from its beginnings in case-mix and resource use theory, to its implementation for payment and now for its current utilization for quality within and outside the hospital. Qual. Manag. Health Care 19, 3–16. doi: 10.1097/QMH.0b013e3181ccbcc3, PMID: 20042929

[ref57] Goldman E. S. Easterling J. Sheiner L. B. (1989). Improving the homogeneity of diagnosis-related groups (DRGs) by using clinical laboratory, demographic, and discharge data. Am. J. Public Health 79, 441–444. doi: 10.2105/AJPH.79.4.441, PMID: 2494894 PMC1349971

[ref58] Goldsmith A. Duggan N. M. Keschner Y. G. Baymon D. M. E. Luo A. D. Nagdev A.. (2025). National Cost Savings from use of artificial intelligence guided echocardiography in the assessment of intermediate-risk patients with Syncope in the emergency department. J. Am. Coll. Emerg. Physic. Open 6:100139. doi: 10.1016/j.acepjo.2025.100139, PMID: 40297198 PMC12035915

[ref59] Gong K. Xue Y. Kong L. Xie X. (2024). Cost prediction for ischemic heart disease hospitalization: interpretable feature extraction using network analysis. J. Biomed. Inform. 154:104652. doi: 10.1016/j.jbi.2024.104652, PMID: 38718897

[ref60] Greenberg J. W. Lancaster T. S. Schuessler R. B. Melby S. J. (2017). Postoperative atrial fibrillation following cardiac surgery: a persistent complication. Eur. J. Cardiothorac. Surg. 52, 665–672. doi: 10.1093/ejcts/ezx039, PMID: 28369234

[ref61] Gudbjartsson T. Helgadottir S. Sigurdsson M. I. Taha A. Jeppsson A. Christensen T. D.. (2020). New-onset postoperative atrial fibrillation after heart surgery. Acta Anaesthesiol. Scand. 64, 145–155. doi: 10.1111/aas.13507, PMID: 31724159

[ref62] Guo H. Li J. Liu H. He J. (2022). Learning dynamic treatment strategies for coronary heart diseases by artificial intelligence: real-world data-driven study. BMC Med. Inform. Decis. Mak. 22:39. doi: 10.1186/s12911-022-01774-0, PMID: 35168623 PMC8845235

[ref63] He H. Zhao S. Xi Y. Ho J. (2023). Meddiff: generating electronic health records using accelerated denoising diffusion model. Arxiv 2302, 1–12. doi: 10.48550/arXiv.2302.04355

[ref64] Henry K. E. Adams R. Parent C. Soleimani H. Sridharan A. Johnson L.. (2022). Factors driving provider adoption of the TREWS machine learning-based early warning system and its effects on sepsis treatment timing. Nat. Med. 28, 1447–1454. doi: 10.1038/s41591-022-01895-z, PMID: 35864251

[ref65] Hughes B. D. Mehta H. B. Sieloff E. Shan Y. Senagore A. J. (2018). DRG migration: a novel measure of inefficient surgical care in a value-based world. Am. J. Surg. 215, 493–496. doi: 10.1016/j.amjsurg.2017.09.035, PMID: 29117915 PMC5837932

[ref66] Islam M. M. Li G. H. Poly T. N. Li Y. J. (2021). Deep DRG: performance of artificial intelligence model for real-time prediction of diagnosis-related groups. Healthcare (Basel) 9:1632. doi: 10.3390/healthcare9121632 34946357 PMC8701302

[ref67] Jafari M. Shoeibi A. Khodatars M. Ghassemi N. Moridian P. Alizadehsani R.. (2023). Automated diagnosis of cardiovascular diseases from cardiac magnetic resonance imaging using deep learning models: a review. Comput. Biol. Med. 160:106998. doi: 10.1016/j.compbiomed.2023.106998, PMID: 37182422

[ref68] Javaid A. Zghyer F. Kim C. Spaulding E. M. Isakadze N. Ding J.. (2022). Medicine 2032: the future of cardiovascular disease prevention with machine learning and digital health technology. Am. J. Prev. Cardiol. 12:100379. doi: 10.1016/j.ajpc.2022.100379, PMID: 36090536 PMC9460561

[ref69] Jayaraman P. Desman J. Sabounchi M. Nadkarni G. N. Sakhuja A. (2024). A primer on reinforcement learning in medicine for clinicians. NPJ Digit. Med. 7:337. doi: 10.1038/s41746-024-01316-0, PMID: 39592855 PMC11599275

[ref70] Joshi S. Sharma M. Das R. P. Rosak-Szyrocka J. Żywiołek J. Muduli K.. (2022). Modeling conceptual framework for implementing barriers of AI in public healthcare for improving operational excellence: experiences from developing countries. Sustainability 14:698. doi: 10.3390/su141811698

[ref71] Kadem M. Garber L. Abdelkhalek M. Al-Khazraji B. K. Keshavarz-Motamed Z. (2023). Hemodynamic modeling, medical imaging, and machine learning and their applications to cardiovascular interventions. IEEE Rev. Biomed. Eng. 16, 403–423. doi: 10.1109/RBME.2022.3142058, PMID: 35015648

[ref72] King Z. Farrington J. Utley M. Kung E. Elkhodair S. Harris S.. (2022). Machine learning for real-time aggregated prediction of hospital admission for emergency patients. NPJ Digit. Med. 5:104. doi: 10.1038/s41746-022-00649-y, PMID: 35882903 PMC9321296

[ref73] Komorowski M. Celi L. A. Badawi O. Gordon A. C. Faisal A. A. (2018). The artificial intelligence clinician learns optimal treatment strategies for sepsis in intensive care. Nat. Med. 24, 1716–1720. doi: 10.1038/s41591-018-0213-5, PMID: 30349085

[ref74] Krittanawong C. Johnson K. W. Rosenson R. S. Wang Z. Aydar M. Baber U.. (2019). Deep learning for cardiovascular medicine: a practical primer. Eur. Heart J. 40, 2058–2073. doi: 10.1093/eurheartj/ehz056, PMID: 30815669 PMC6600129

[ref75] Kutz A. Gut L. Ebrahimi F. Wagner U. Schuetz P. Mueller B. (2019). Association of the Swiss Diagnosis-Related Group Reimbursement System with Length of stay, mortality, and readmission rates in hospitalized adult patients. JAMA Netw. Open 2:e188332. doi: 10.1001/jamanetworkopen.2018.8332, PMID: 30768196 PMC6484617

[ref76] Kwak M. J. Chang M. Chiadika S. Aguilar D. Avritscher E. Deshmukh A.. (2022). Healthcare expenditure associated with polypharmacy in older adults with cardiovascular diseases. Am. J. Cardiol. 169, 156–158. doi: 10.1016/j.amjcard.2022.01.012, PMID: 35168755 PMC9313779

[ref77] Laghari A. A. Sun Y. Alhussein M. Aurangzeb K. Anwar M. S. Rashid M. (2023). Deep residual-dense network based on bidirectional recurrent neural network for atrial fibrillation detection. Sci. Rep. 13:15109. doi: 10.1038/s41598-023-40343-x, PMID: 37704659 PMC10499947

[ref78] Lee S. H. Kim M. K. Rhee E. J. (2020). Effects of cardiovascular risk factor variability on health outcomes. Endocrinol. Metab. 35, 217–226. doi: 10.3803/EnM.2020.35.2.217, PMID: 32615706 PMC7386100

[ref79] Lee S. W. Lee H. C. Suh J. Lee K. H. Lee H. Seo S.. (2022). Multi-center validation of machine learning model for preoperative prediction of postoperative mortality. NPJ Digit. Med. 5:91. doi: 10.1038/s41746-022-00625-6, PMID: 35821515 PMC9276734

[ref80] Li B. Eisenberg N. Beaton D. Lee D. S. Al-Omran L. Wijeysundera D. N. (2024). Using machine learning to predict outcomes following transfemoral carotid artery stenting. J. Am. Heart Assoc. 13:e035425. doi: 10.1161/JAHA.124.035425, PMID: 39189482 PMC11646515

[ref81] Li Q. Fan X. Jian W. (2023). Impact of diagnosis-related-group (DRG) payment on variation in hospitalization expenditure: evidence from China. BMC Health Serv. Res. 23:688. doi: 10.1186/s12913-023-09686-z, PMID: 37355657 PMC10290781

[ref82] Liu J. Capurro D. Nguyen A. Verspoor K. (2021). Early prediction of diagnostic-related groups and estimation of hospital cost by processing clinical notes. NPJ Digit Med 4:103. doi: 10.1038/s41746-021-00474-9, PMID: 34211109 PMC8249417

[ref83] Liu B. Chang H. Yang D. Yang F. Wang Q. Deng Y.. (2023). A deep learning framework assisted echocardiography with diagnosis, lesion localization, phenogrouping heterogeneous disease, and anomaly detection. Sci. Rep. 13:3. doi: 10.1038/s41598-022-27211-w, PMID: 36593284 PMC9807607

[ref84] Liu W. C. Lin C. Lin C.-S. Tsai M. C. Chen S. J. Tsai S.-H.. (2021). An artificial intelligence-based alarm strategy facilitates management of acute myocardial infarction. J. Pers. Med. 11:1149. doi: 10.3390/jpm11111149, PMID: 34834501 PMC8623357

[ref85] Liu S. See K. C. Ngiam K. Y. Celi L. A. Sun X. Feng M. (2020). Reinforcement learning for clinical decision support in critical care: comprehensive review. J. Med. Internet Res. 22:e18477. doi: 10.2196/18477, PMID: 32706670 PMC7400046

[ref86] Liu C. Xia Y. Qiu J. (2014). Influence of fill in of homepage of medical record on diagnosis-related groups data quality. Chinese Med. Rec. 1, 467–470. doi: 10.3109/23256176.2013.877696

[ref87] Lu X. H. Liu A. Fuh S. C. Lian Y. Guo L. Yang Y.. (2021). Recurrent disease progression networks for modelling risk trajectory of heart failure. PLoS One 16:e0245177. doi: 10.1371/journal.pone.0245177, PMID: 33406155 PMC7787457

[ref88] Lundberg S. M. Nair B. Vavilala M. S. Horibe M. Eisses M. J. Adams T.. (2018). Explainable machine-learning predictions for the prevention of hypoxaemia during surgery. Nat Biomed Eng 2, 749–760. doi: 10.1038/s41551-018-0304-0, PMID: 31001455 PMC6467492

[ref89] Lundervold A. S. Lundervold A. (2019). An overview of deep learning in medical imaging focusing on MRI. Z. Med. Phys. 29, 102–127. doi: 10.1016/j.zemedi.2018.11.002, PMID: 30553609

[ref90] Luo Y. Liu Z. Liu Q.. Deep stable representation learning on electronic health records. 2022 IEEE international conference on data mining (ICDM) (2022) 1077–1082.

[ref91] Maleki Varnosfaderani S. Forouzanfar M. (2024). The role of AI in hospitals and clinics: transforming healthcare in the 21st century. Bioengineering 11:337. doi: 10.3390/bioengineering11040337, PMID: 38671759 PMC11047988

[ref92] Marafino B. J. Escobar G. J. Baiocchi M. T. Liu V. X. Plimier C. C. Schuler A. (2021). Evaluation of an intervention targeted with predictive analytics to prevent readmissions in an integrated health system: observational study. BMJ 374:1747. doi: 10.1136/bmj.n1747, PMID: 34380667 PMC8356037

[ref93] Mehra T. Muller C. T. Volbracht J. Seifert B. Moos R. (2015). Predictors of high profit and high deficit outliers under Swiss DRG of a tertiary care center. PLoS One 10:e0140874. doi: 10.1371/journal.pone.0140874, PMID: 26517545 PMC4627843

[ref94] Midhun J. Arun Raj A. S. Beereddy M. Gandu S. P. Sudha G. P. Gandu B. H.. Ensemble deep learning models for accurate prediction of cardiovascular disease risk: a comparative analysis. 2023 2nd international conference on edge computing and applications (ICECAA); (2023). 1002–1007.

[ref95] Mihailovic N. Kocic S. Jakovljevic M. (2016). Review of diagnosis-related group-based financing of hospital care. Health Serv. Res. Manag. Epidemiol. 3:2333392816647892. doi: 10.1177/2333392816647892, PMID: 28462278 PMC5266471

[ref96] Milstein R. Schreyogg J. (2024). The end of an era? Activity-based funding based on diagnosis-related groups: a review of payment reforms in the inpatient sector in 10 high-income countries. Health Policy 141:104990. doi: 10.1016/j.healthpol.2023.104990, PMID: 38244342

[ref97] Miranda A. S. Rocha G. B. F. Neto O. P. Santos L. D. R. Ferreira M. B. G. Magnabosco P. (2021). Associations between surgical wound infectious and clinical profile in patients undergoing cardiac surgery. Am. J. Cardiovasc. Dis. 11, 231–238. 34084658 PMC8166584

[ref98] Mittermaier M. Raza M. Kvedar J. C. (2023). Collaborative strategies for deploying AI-based physician decision support systems: challenges and deployment approaches. NPJ Digit. Med. 6:137. doi: 10.1038/s41746-023-00889-6, PMID: 37543707 PMC10404285

[ref99] Moradi A. Olanisa O. O. Nzeako T. Shahrokhi M. Esfahani E. Fakher N.. (2024). Revolutionizing cardiac imaging: a scoping review of artificial intelligence in echocardiography, CTA, and cardiac MRI. J Imaging 10:193. doi: 10.3390/jimaging10080193, PMID: 39194982 PMC11355719

[ref100] Nagarajan V. D. Lee S. L. Robertus J. L. Nienaber C. A. Trayanova N. A. Ernst S. (2021). Artificial intelligence in the diagnosis and management of arrhythmias. Eur. Heart J. 42, 3904–3916. doi: 10.1093/eurheartj/ehab544, PMID: 34392353 PMC8497074

[ref101] Naidu J. N. Varma M. A. Madhuri P. S. Shankar D. Matta D. S. Ramya S.. Enhancing heart disease prediction through a heterogeneous ensemble DL models. International Conference on Cognitive Computing and Cyber Physical Systems; (2024) 58–73.

[ref102] Nasarudin N. A. Al Jasmi F. Sinnott R. O. Zaki N. Al Ashwal H. Mohamed E. A.. (2024). A review of deep learning models and online healthcare databases for electronic health records and their use for health prediction. Artif. Intell. Rev. 57:876. doi: 10.1007/s10462-024-10876-2

[ref103] Ninomiya K. Kageyama S. Shiomi H. Kotoku N. Masuda S. Revaiah P. C.. (2023). Can machine learning aid the selection of percutaneous vs surgical revascularization? J. Am. Coll. Cardiol. 82, 2113–2124. doi: 10.1016/j.jacc.2023.09.818, PMID: 37993203

[ref104] Obermeyer Z. Powers B. Vogeli C. Mullainathan S. (2019). Dissecting racial bias in an algorithm used to manage the health of population. Sci. Transl. Med. 366, 447–453. doi: 10.1126/science.aax2342 31649194

[ref105] Pavuluri S. Sangal R. Sather J. Taylor R. A. (2024). Balancing act: the complex role of artificial intelligence in addressing burnout and healthcare workforce dynamics. BMJ Health Care Inform. 31:120. doi: 10.1136/bmjhci-2024-101120, PMID: 39181545 PMC11344516

[ref106] Pokaprakarn T. Kitzmiller R. R. Moorman J. R. Lake D. E. Krishnamurthy A. K. Kosorok M. R. (2022). Sequence to sequence ECG cardiac rhythm classification using convolutional recurrent neural networks. IEEE J. Biomed. Health Inform. 26, 572–580. doi: 10.1109/JBHI.2021.3098662, PMID: 34288883 PMC9033271

[ref107] Pomponio R. Erus G. Habes M. Doshi J. Srinivasan D. Mamourian E.. (2020). Harmonization of large MRI datasets for the analysis of brain imaging patterns throughout the lifespan. NeuroImage 208:116450. doi: 10.1016/j.neuroimage.2019.116450, PMID: 31821869 PMC6980790

[ref108] Pyrros A. Rodriguez Fernandez J. Borstelmann S. M. Flanders A. Wenzke D. Hart E.. (2022). Validation of a deep learning, value-based care model to predict mortality and comorbidities from chest radiographs in COVID-19. PLoS Digit. Health 1:e0000057. doi: 10.1371/journal.pdig.0000057, PMID: 36812559 PMC9931278

[ref109] Rabbani N. Ma S. P. Li R. C. Winget M. Weber S. Boosi S.. (2023). Targeting repetitive laboratory testing with electronic health records-embedded predictive decision support: a pre-implementation study. Clin. Biochem. 113, 70–77. doi: 10.1016/j.clinbiochem.2023.01.002, PMID: 36623759 PMC9936847

[ref110] Rai H. M. Chatterjee K. (2021). Hybrid CNN-LSTM deep learning model and ensemble technique for automatic detection of myocardial infarction using big ECG data. Appl. Intell. 52, 5366–5384. doi: 10.1007/s10489-021-02696-6

[ref111] Rai H. M. Chatterjee K. Mukherjee C. Hybrid CNN-LSTM model for automatic prediction of cardiac arrhythmias from ECG big data. 2020 IEEE 7th Uttar Pradesh section international conference on electrical, electronics and computer engineering (UPCON); (2020). p. 1–6.

[ref112] Rajashekar N. C. Shin Y. E. Pu Y. Chung S. You K. Giuffre M.. Human-algorithmic interaction using a large language model-augmented artificial intelligence clinical decision support system. Proceedings of the 2024 CHI Conference on Human Factors in Computing Systems; (2024). p. 1–20.

[ref113] Rajkomar A. Dean J. Kohane I. (2019). Machine learning in medicine. N. Engl. J. Med. 380, 1347–1358. doi: 10.1056/NEJMra1814259, PMID: 30943338

[ref114] Rajkomar A. Hardt M. Howell M. D. Corrado G. Chin M. H. (2018). Ensuring fairness in machine learning to advance health equity. Ann. Intern. Med. 169, 866–872. doi: 10.7326/M18-1990, PMID: 30508424 PMC6594166

[ref115] Rajpurkar P. Hannun A. Y. Haghpanahi M. Bourn C. Ng A. Y. (2017). Cardiologist-level arrhythmia detection with convolutional neural networks. Arxiv 1707, 1–9. doi: 10.48550/arXiv.1707.01836

[ref116] Ravi D. Wong C. Deligianni F. Berthelot M. Andreu-Perez J. Lo B.. (2017). Deep learning for health informatics. IEEE J. Biomed. Health Inform. 21, 4–21. doi: 10.1109/JBHI.2016.2636665 28055930

[ref117] Rieke N. Hancox J. Li W. Milletarì F. Roth H. R. Albarqouni S.. (2020). The future of digital health with federated learning. NPJ Digit. Med. 3:119. doi: 10.1038/s41746-020-00323-1, PMID: 33015372 PMC7490367

[ref118] Roth G. A. Mensah G. A. Johnson C. O. Addolorato G. Ammirati E. Baddour L. M.. (2020). Global burden of cardiovascular diseases and risk factors, 1990-2019: update from the GBD 2019 study. J. Am. Coll. Cardiol. 76, 2982–3021. doi: 10.1016/j.jacc.2020.11.010, PMID: 33309175 PMC7755038

[ref119] Ruan Y. Lan X. Tan D. J. Abdullah H. R. Feng M. (2023). P-transformer: a prompt-based multimodal transformer architecture for medical tabular data. Arxiv 2303, 1–13.

[ref120] Sabe S. A. Sabe M. A. Kennedy K. F. Sellke F. W. Ehsan A. (2023). Risk factors for heart failure readmission after cardiac surgery. JACC Adv. 2:100599. doi: 10.1016/j.jacadv.2023.100599, PMID: 38938350 PMC11198058

[ref121] Sadr H. Salari A. Ashoobi M. T. Nazari M. (2024). Cardiovascular disease diagnosis: a holistic approach using the integration of machine learning and deep learning models. Eur. J. Med. Res. 29:455. doi: 10.1186/s40001-024-02044-7, PMID: 39261891 PMC11389500

[ref122] Sazdov B. Tashkovska M. Krsteski S. Jovanovski B. Kalabakov S. Rakovic V.. Prediction of hospital readmission using federated learning. (2023) 30th International Conference on Systems, Signals and Image Processing (IWSSIP). p. 1–5.

[ref123] Schiano C. Benincasa G. Franzese M. Della Mura N. Pane K. Salvatore M.. (2020). Epigenetic-sensitive pathways in personalized therapy of major cardiovascular diseases. Pharmacol. Ther. 210:107514. doi: 10.1016/j.pharmthera.2020.107514, PMID: 32105674

[ref124] Schweizer M. L. Chiang H. Y. Septimus E. Moody J. Braun B. Hafner J.. (2015). Association of a bundled intervention with surgical site infections among patients undergoing cardiac, hip, or knee surgery. JAMA 313, 2162–2171. doi: 10.1001/jama.2015.5387, PMID: 26034956

[ref125] Sendak M. Elish M. C. Gao J. Futoma J. Ratliff W. Nichols M.. "The human body is a black box". Proceedings of the 2020 conference on fairness, accountability, and transparency; (2020). p. 99–109.

[ref126] Shah P. Kendall F. Khozin S. Goosen R. Hu J. Laramie J.. (2019). Artificial intelligence and machine learning in clinical development: a translational perspective. NPJ Digit. Med. 2:69. doi: 10.1038/s41746-019-0148-3, PMID: 31372505 PMC6659652

[ref127] Shameer K. Johnson K. W. Glicksberg B. S. Dudley J. T. Sengupta P. P. (2018). Machine learning in cardiovascular medicine: are we there yet? Heart 104, 1156–1164. doi: 10.1136/heartjnl-2017-311198, PMID: 29352006

[ref128] Shetty M. K. Kunal S. Girish M. Girish M. P. Qamar A. Arora S.. (2022). Machine learning based model for risk prediction after ST-elevation myocardial infarction: insights from the North India ST elevation myocardial infarction (NORIN-STEMI) registry. Int. J. Cardiol. 362, 6–13. doi: 10.1016/j.ijcard.2022.05.023, PMID: 35577162

[ref129] Shickel B. Loftus T. J. Ruppert M. Upchurch G. R. Jr. Ozrazgat-Baslanti T. Rashidi P.. (2023). Dynamic predictions of postoperative complications from explainable, uncertainty-aware, and multi-task deep neural networks. Sci. Rep. 13:1224. doi: 10.1038/s41598-023-27418-5, PMID: 36681755 PMC9867692

[ref130] Smith M. Sattler A. Hong G. Lin S. (2021). From code to bedside: implementing artificial intelligence using quality improvement methods. J. Gen. Intern. Med. 36, 1061–1066. doi: 10.1007/s11606-020-06394-w, PMID: 33469745 PMC8041947

[ref131] Song Y. Ren S. Lu Y. Fu X. Wong K. K. L. (2022). Deep learning-based automatic segmentation of images in cardiac radiography: a promising challenge. Comput. Methods Prog. Biomed. 220:106821. doi: 10.1016/j.cmpb.2022.106821 35487181

[ref132] Spadaccio C. Benedetto U. (2018). Coronary artery bypass grafting (CABG) vs. percutaneous coronary intervention (PCI) in the treatment of multivessel coronary disease: quo vadis?-a review of the evidences on coronary artery disease. Ann. Cardiothorac. Surg. 7, 506–515. doi: 10.21037/acs.2018.05.17, PMID: 30094215 PMC6082779

[ref133] Srivastava D. Henschke C. Virtanen L. Lotman E.-M. Friebel R. Ardito V.. (2023). Promoting the systematic use of real-world data and real-world evidence for digital health technologies across Europe: a consensus framework. Health Econ. Policy Law 18, 395–410. doi: 10.1017/S1744133123000208, PMID: 37705236

[ref134] Stogiannos N. Gillan C. Precht H. Reis C. Kumar A. O'Regan T.. (2024). A multidisciplinary team and multiagency approach for AI implementation: a commentary for medical imaging and radiotherapy key stakeholders. J. Med. Imaging Radiat. Sci. 55:101717. doi: 10.1016/j.jmir.2024.101717, PMID: 39067309

[ref135] Sudha V. K. Kumar D. (2023). Hybrid CNN and LSTM network for heart disease prediction. SN Comput. Sci. 4:598. doi: 10.1007/s42979-022-01598-9

[ref136] Sun A. Hong W. Li J. Mao J. (2024). An arrhythmia classification model based on a CNN-LSTM-SE algorithm. Sensors 24:306. doi: 10.3390/s24196306, PMID: 39409344 PMC11478372

[ref137] Talaat F. M. (2024). Revolutionizing cardiovascular health: integrating deep learning techniques for predictive analysis of personal key indicators in heart disease. Neural Comput. Applic. 37, 1–24. doi: 10.1007/s00521-024-10453-2

[ref138] Tariq A. Lancaster L. Elugunti P. Siebeneck E. Noe K. Borah B.. (2023). Graph convolutional network-based fusion model to predict risk of hospital acquired infections. J. Am. Med. Inform. Assoc. 30, 1056–1067. doi: 10.1093/jamia/ocad045, PMID: 37027831 PMC10198521

[ref139] Varani E. Balducelli M. Gatti C. Tesselli M. R. Vecchi G. Maresta A. (2005). Cost of single-vessel and multivessel coronary drug-eluting stenting: comparison to the DRG funding level. Ital. Heart J. 6, 52–58. 15773274

[ref140] Verma A. Sanaiha Y. Hadaya J. Maltagliati A. J. Tran Z. Ramezani R.. (2022). Parsimonious machine learning models to predict resource use in cardiac surgery across a statewide collaborative. JTCVS Open 11, 214–228. doi: 10.1016/j.xjon.2022.04.017, PMID: 36172420 PMC9510828

[ref141] Virani S. S. Alonso A. Aparicio H. J. Benjamin E. J. Bittencourt M. S. Callaway C. W.. (2021). Heart disease and stroke Statistics-2021 update: a report from the American Heart Association. Circulation 143, e254–e743. doi: 10.1161/CIR.0000000000000950, PMID: 33501848 PMC13036842

[ref142] Wang Y. Pillai M. Zhao Y. Curtin C. Hernandez-Boussard T. (2024). Fair EHR-CLP: towards fairness-aware clinical predictions with contrastive learning in multimodal electronic health records. Arxiv 252, 1–26. doi: 10.48550/arXiv.2402.00955

[ref143] Wang K. Zhang K. Liu B. Chen W. Han M. (2024). Early prediction of sudden cardiac death risk with nested LSTM based on electrocardiogram sequential features. BMC Med. Inform. Decis. Mak. 24:94. doi: 10.1186/s12911-024-02493-4, PMID: 38600479 PMC11005267

[ref144] Wang L. Zhang Z. Wang D. Cao W. Zhou X. Zhang P.. (2023). Human-centered design and evaluation of AI-empowered clinical decision support systems: a systematic review. Front. Comput. Sci. 5:1187299. doi: 10.3389/fcomp.2023.1187299

[ref145] Weir-McCall J. R. Williams M. C. Shah A. S. V. Roditi G. Rudd J. H. F. Newby D. E.. (2023). National Trends in coronary artery disease imaging: associations with health care outcomes and costs. JACC Cardiovasc. Imaging 16, 659–671. doi: 10.1016/j.jcmg.2022.10.022, PMID: 36752441

[ref146] Wolff J. Pauling J. Keck A. Baumbach J. (2021). Success factors of artificial intelligence implementation in healthcare. Front. Digit. Health 3:594971. doi: 10.3389/fdgth.2021.594971, PMID: 34713083 PMC8521923

[ref147] Wu M. Du X. Gu R. Wei J. (2021). Artificial intelligence for clinical decision support in Sepsis. Front. Med. 8:665464. doi: 10.3389/fmed.2021.665464, PMID: 34055839 PMC8155362

[ref148] Xiao C. Ma T. Dieng A. B. Blei D. M. Wang F. (2018). Readmission prediction via deep contextual embedding of clinical concepts. PLoS One 13:e0195024. doi: 10.1371/journal.pone.0195024, PMID: 29630604 PMC5890980

[ref149] Xu Y. Zhang J. Gu Y. (2024). Privacy-preserving heterogeneous federated learning for sensitive healthcare data. Arxiv 2406, 1–7.

[ref150] Yang C. Hu X. Zhu Q. Tu Q. Geng H. Xu J.. (2024). Individual medical costs prediction methods based on clinical notes and DRGs. IEEE J. Radio Freq. Identif. 8, 412–418. doi: 10.1109/JRFID.2024.3392682

[ref151] Yom-Tov E. Feraru G. Kozdoba M. Mannor S. Tennenholtz M. Hochberg I. (2017). Encouraging physical activity in patients with diabetes: intervention using a reinforcement learning system. J. Med. Internet Res. 19:e338. doi: 10.2196/jmir.7994, PMID: 29017988 PMC5654735

[ref152] Yu Z. Li Y. Kim J. C. Huang K. Luo Y. Wang M. (2023). Deep reinforcement learning for cost-effective medical diagnosis. Arxiv 2302, 1–25. doi: 10.48550/arXiv.2302.10261

[ref153] Yu M. Y. Son Y. J. (2024). Machine learning-based 30-day readmission prediction models for patients with heart failure: a systematic review. Eur. J. Cardiovasc. Nurs. 23, 711–719. doi: 10.1093/eurjcn/zvae031, PMID: 38421187

[ref154] Yuan S. Liu W. Wei F. Zhang H. Wang S. Zhu W.. (2019). Impacts of hospital payment based on diagnosis related groups (DRGs) with global budget on resource use and quality of care: a case study in China. Iran. J. Public Health 48, 238–246. 31205877 PMC6556178

[ref155] Zhao Y. Zheng X. Wang J. Xu D. Li S. Lv J.. (2021). Effect of clinical decision support systems on clinical outcome for acute kidney injury: a systematic review and meta-analysis. BMC Nephrol. 22:271. doi: 10.1186/s12882-021-02459-y, PMID: 34348688 PMC8335454

[ref156] Zhu Y. Swanson K. M. Rojas R. L. Wang Z. Sauver J. L. Visscher S. L.. (2020). Systematic review of the evidence on the cost-effectiveness of pharmacogenomics-guided treatment for cardiovascular diseases. Genet. Med. 22, 475–486. doi: 10.1038/s41436-019-0667-y, PMID: 31591509 PMC7056639

[ref157] Zou K. Li H. Y. Zhou D. Liao Z. J. (2020). The effects of diagnosis-related groups payment on hospital healthcare in China: a systematic review. BMC Health Serv. Res. 20:112. doi: 10.1186/s12913-020-4957-5, PMID: 32050962 PMC7017558

